# Comparison of Four Active SARS-CoV-2 Surveillance Strategies in Representative Population Sample Points: Two-Factor Factorial Randomized Controlled Trial

**DOI:** 10.2196/44204

**Published:** 2023-08-17

**Authors:** Andreas Deckert, Simon Anders, Ivonne Morales, Manuela De Allegri, Hoa Thi Nguyen, Aurélia Souares, Shannon McMahon, Matthias Meurer, Robin Burk, Dan Lou, Lucia Brugnara, Matthias Sand, Lisa Koeppel, Lena Maier-Hein, Tobias Ross, Tim J Adler, Stephan Brenner, Christopher Dyer, Konrad Herbst, Svetlana Ovchinnikova, Michael Marx, Paul Schnitzler, Michael Knop, Till Bärnighausen, Claudia M Denkinger

**Affiliations:** 1 Heidelberg Institute of Global Health Heidelberg Germany; 2 Center for Molecular Biology Heidelberg Heidelberg Germany; 3 Division of Infectious Disease and Tropical Medicine, Heidelberg University Hospital Heidelberg Germany; 4 evaplan GmbH at the University Hospital Heidelberg Germany; 5 GESIS Leibniz-Institute for the Social Sciences Mannheim Germany; 6 Division of Computer Assisted Medical Interventions, German Cancer Research Centre Heidelberg Germany; 7 Gesundheitsamt des Rhein-Neckar-Kreises Heidelberg Germany; 8 Center of Infectious Diseases, Virology, Heidelberg University Hospital Heidelberg Germany

**Keywords:** COVID-19, SARS-CoV-2, randomized controlled trial, multiarm, cluster sampling, surveillance, effectiveness, pandemic

## Abstract

**Background:**

The COVID-19 pandemic is characterized by rapid increases in infection burden owing to the emergence of new variants with higher transmissibility and immune escape. To date, monitoring the COVID-19 pandemic has mainly relied on passive surveillance, yielding biased epidemiological measures owing to the disproportionate number of undetected asymptomatic cases. Active surveillance could provide accurate estimates of the true prevalence to forecast the evolution of the pandemic, enabling evidence-based decision-making.

**Objective:**

This study compared 4 different approaches of active SARS-CoV-2 surveillance focusing on feasibility and epidemiological outcomes.

**Methods:**

A 2-factor factorial randomized controlled trial was conducted in 2020 in a German district with 700,000 inhabitants. The epidemiological outcome comprised SARS-CoV-2 prevalence and its precision. The 4 study arms combined 2 factors: individuals versus households and direct testing versus testing conditioned on symptom prescreening. Individuals aged ≥7 years were eligible. Altogether, 27,908 addresses from 51 municipalities were randomly allocated to the arms and 15 consecutive recruitment weekdays. Data collection and logistics were highly digitized, and a website in 5 languages enabled low-barrier registration and tracking of results. Gargle sample collection kits were sent by post. Participants collected a gargle sample at home and mailed it to the laboratory. Samples were analyzed with reverse transcription loop-mediated isothermal amplification (RT-LAMP); positive and weak results were confirmed with real-time reverse transcription–polymerase chain reaction (RT-PCR).

**Results:**

Recruitment was conducted between November 18 and December 11, 2020. The response rates in the 4 arms varied between 34.31% (2340/6821) and 41.17% (2043/4962). The prescreening classified 16.61% (1207/7266) of the patients as COVID-19 symptomatic. Altogether, 4232 persons without prescreening and 7623 participating in the prescreening provided 5351 gargle samples, of which 5319 (99.4%) could be analyzed. This yielded 17 confirmed SARS-CoV-2 infections and a combined prevalence of 0.36% (95% CI 0.14%-0.59%) in the arms without prescreening and 0.05% (95% CI 0.00%-0.108%) in the arms with prescreening (initial contacts only). Specifically, we found a prevalence of 0.31% (95% CI 0.06%-0.58%) for individuals and 0.35% (95% CI 0.09%-0.61%) for households, and lower estimates with prescreening (0.07%, 95% CI 0.0%-0.15% for individuals and 0.02%, 95% CI 0.0%-0.06% for households). Asymptomatic infections occurred in 27% (3/11) of the positive cases with symptom data. The 2 arms without prescreening performed the best regarding effectiveness and accuracy.

**Conclusions:**

This study showed that postal mailing of gargle sample kits and returning home-based self-collected liquid gargle samples followed by high-sensitivity RT-LAMP analysis is a feasible way to conduct active SARS-CoV-2 population surveillance without burdening routine diagnostic testing. Efforts to improve participation rates and integration into the public health system may increase the potential to monitor the course of the pandemic.

**Trial Registration:**

Deutsches Register Klinischer Studien (DRKS) DRKS00023271; https://tinyurl.com/3xenz68a

**International Registered Report Identifier (IRRID):**

RR2-10.1186/s13063-021-05619-5

## Introduction

### Background

Epidemiological surveillance of populations using serological or nucleic acid–based diagnostics is a well-known public health preparedness tool and is, for example, implemented in the global influenza surveillance network. Here, a system of World Health Organization collaborating centers acts mainly reactively to seasonally adjust influenza vaccines depending on emerging variants in representative samples [1,2]. In contrast, the COVID-19 pandemic has been characterized by less seasonality and a comparatively faster periodicity of episodes in which the burden of infection in the population rapidly increased because of the emergence of new variants with higher transmissibility and immune escape. The ever-present threat of overburdening the health care systems resulted in the implementation of containment measures based on limited forecasting tools [3].

Both presymptomatic and asymptomatic SARS-CoV-2 carriers are able to transmit; thus, the proportion of asymptomatic cases plays a key role in the spread of the virus, challenging the containment of this highly contagious disease [4-7]. With respect to the SARS-CoV-2 alpha variant (B.1.1.7, prevalent at the time of the study), asymptomatic or minimally symptomatic SARS-CoV-2 carriers accounted for approximately 40% of all unvaccinated infected individuals [8]. A surveillance system that randomly samples a representative population to test for active infection is therefore needed for pandemic preparedness. If combined with longitudinal serological studies to monitor historical exposure and thus the extent of immunity, the prevalence estimates could be incorporated into a prognostic model to effectively monitor the pandemic and predict the further course of the outbreak. This would enable evidence-based decision-making, especially as strict lockdowns and curfews can only serve as temporary measures [9]. Furthermore, active disease surveillance could assess symptomatology in combination with downstream genome sequencing [10].

Most countries mainly used results from different testing purposes in the so-called passive surveillance as a surrogate for active systems to restrictedly monitor the course of the pandemic [11,12]. This involves recording persons presenting to the health system with COVID-19–like symptoms to initiate their treatment, as well as symptomatically and asymptomatically infected persons identified through targeted screening (eg, workplaces and retirement homes), supplemented by contacts of detected cases who tested positive with the aim of isolating these individuals and containing the spread of the infection [13]. Others extended existing primary care influenza surveillance systems to virological and serological SARS-CoV-2 surveillance [14]. However, these largely symptom-driven surveillance systems do not provide representative prevalence estimates and are therefore of limited use in terms of informing decision makers [11]. Similarly, surveillance studies that rely on syndromic surveillance rather than direct testing focus on symptomatic cases only and are further limited as they do not differentiate between respiratory pathogens [15,16]. In contrast, active surveillance would test a representative proportion of the population without necessarily assuming the presence of COVID-19 symptoms [17].

However, testing for SARS-CoV-2 for surveillance in communities requires extensive, complex testing capacities and different strategies compared with testing for individual health purposes and screening with respect to sampling, turnaround time, and reporting [18,19]. Real-time reverse transcription–polymerase chain reaction (RT-PCR), a laboratory technology used to detect viral RNA, is considered the gold standard for SARS-CoV-2 detection during acute infection and up to 2 to 3 weeks thereafter [20]. However, RT-PCR is costly and only applicable in population screening if pooling techniques are applied [21,22]. Alternatively, reverse transcription loop-mediated isothermal amplification (RT-LAMP)–based method allows for the detection of viral RNA with sensitivity similar to that of RT-PCR but with reduced cost and straightforward application, not requiring a thermal cycler, hence enabling scale-up testing [23-26].

In addition, there is sufficient evidence that saliva, gargle solution, and expectorated mucus are suitable sources of biological material for the sensitive detection of SARS-CoV-2 [27-32]. Saliva samples are very stable as their viral load hardly changes even over several days at room temperature (20-25 °C), which makes it easily transportable even by post [33]. Therefore, the combination of self-collected saliva samples shipped by post and analyzed by RT-LAMP appears to be a promising approach for active population surveillance.

However, there is limited evidence regarding the use of comprehensive and effective active surveillance strategies. Most of the active surveillance studies conducted in the general population have exhibited a repeated cross-sectional design. To date, the best-described active surveillance systems of similar type have been implemented in the United Kingdom using RT-PCR assays and self-administered swabs [19,34]. These 2 attempts have demonstrated the importance and viability of implementing active surveillance for SARS-CoV-2 in affluent areas by providing significant SARS-COV-2 prevalence estimates in the community during various time points. Some studies have observed fixed cohorts over time [35,36]. Although these studies have shown the feasibility of active surveillance, no study to date has compared different surveillance approaches to assess their comparative feasibility, effectiveness, and cost. Nonetheless, the combination of these factors is relevant to public health decision makers facing resource constraints, with testing capacity depending mainly on external factors (eg, production of reagents and test materials), independent of the testing strategy.

The question for decision makers in health policy is therefore which active surveillance strategy is most effective in monitoring the actual progress of an epidemic or pandemic with a limited number of available tests and without limiting the capacity of passive surveillance.

### Objectives

The objective of this study was to compare 4 different approaches to population-level surveillance of active SARS-CoV-2 infection and to report the epidemiological outcomes and feasibility. The cost-effectiveness was reported separately [37].

## Methods

This study followed the principles of CONSORT (Consolidated Standards of Reporting Trials; extension for multiarm parallel-group randomized trials) statement (Multimedia Appendix 1) [38]. Details regarding the methods and study design are provided in the study protocol by Deckert et al [39].

### Ethics Approval

The trial was approved by the ethics committee at the University of Heidelberg on November 2, 2020 (amendment November 9, 2020; file number S790/2020).

### Participation and Informed Consent

The study applied the Declaration of Helsinki ethical standards and adhered to the legal requirements for research on humans in Germany as stated in the guidelines for good clinical practice and the Medical Devices Act (Medizinproduktegesetz) issued by the Ministry of Health of Germany and implemented by the Federal Institute for Drugs and Medical Devices.

With the invitation letter, participants were informed about the study purpose and procedures in an age group–appropriate manner by means of a detailed brochure; all web-based information was also available in 5 languages. Children and adolescents were asked to assent their participation with a mandatorily complemented consent of their legal representatives. Consent or assent to participate could be given on the web or on paper, whereas additional consent or assent to get the gargle sample analyzed had to be provided on paper (Multimedia Appendix 2 [20-26,28-33,39-48]). Participants’ addresses were provided by the registers of residents based on a German law (Bundesmeldegesetz, § 46 Gruppenauskunft) that allows research institutions to draw collective address data for research purposes; consent was sought from the population register offices.

Data protection measures were implemented according to the Basic Data Protection Regulation (article 6, paragraph 1, letter a Datenschutz-Grundverordnung) and the Federal Data Protection Act. The manner of data collection and scope and content of data collected were authorized by the data protection officer of Heidelberg University. The consent forms provided information about the data protection measures and pseudonymization of data to identify individuals who tested positive; data analyses were conducted with anonymized data only. Withdrawal from study participation and deletion of data were possible at any time during the study. Participants were informed that the data could be used beyond the study for other research purposes related to SARS-CoV-2 or other respiratory viruses; however, the gargle samples were destroyed after study completion.

Compensation for study participation was not provided.

### Trial Registration

The trial was registered (November 30, 2020) on the German Clinical Trials Register (registration number DRKS00023271), and the study protocol has been published accordingly [49].

### Study Design

The study was designed as a 2-factor factorial, multiarm parallel randomized controlled trial. The 4 study arms represented all combinations of two factors: i) testing unconditional (A) versus testing under the condition of upstream COVID-19 symptom prescreening (B), and ii) testing individuals (1) versus households (2). The designation of the 4 study arms A1, A2, B1, and B2 depict these combinations.

Individuals aged ≥7 years who consented (by legal guardian in the case of minors) were eligible and provided a self-collected gargle sample (all 4 study arms) after completion of a prescreening questionnaire (B1 and B2).

### Study Setting

The trial was conducted during the second SARS-CoV-2 wave in fall 2020 in Germany in Heidelberg and the Rhine-Neckar district, which is the location of Heidelberg University and the catchment area of the closely cooperating district health authority. The public health authority of the Rhine-Neckar district, also responsible for Heidelberg city, is responsible for 56 municipalities with approximately 700,000 inhabitants. Containment measures were tightened by mid-December 2020, and vaccination was not yet available at that time.

### Outcomes

We considered effectiveness, feasibility, and costs to comprehensively inform policy makers as trade-offs are to be expected when the evidence is reviewed by an evidence-to-decision framework, for example, Grading of Recommendations, Assessment, Development, and Evaluations [49]. Here, we report the feasibility and epidemiological outcomes and review the cost-effectiveness separately. The epidemiological outcomes included the following: (1) cumulative SARS-CoV-2 prevalence and (2) prevalence precision.

The secondary outcomes were the participation rate (number of gargle samples; A1 and A2), the prescreening results or gargle samples (B1 and B2) divided by the number of contacts, the population prevalence estimation, and the symptom frequencies.

### Sample Size

As high-quality test capacity is a limiting resource in active surveillance, we designed the study so that each arm would end up with the same number of laboratory tests, competing for feasibility, effectiveness, and cost-effectiveness. However, a composite end point was not possible, and a priori assumptions regarding cost-effectiveness could only be made arbitrarily. Therefore, we powered the effectiveness part to finally receive the same number of gargle samples in each arm to estimate a specific prevalence with a certain precision, based on the estimator theoretically closest to the true prevalence in study arm A1 (randomly selected individuals). The costs required to achieve this equal number of gargle samples might differ by design in the study arms, as may the number of positive cases among the gargle samples. Strategy A2 was designed to account for the likelihood of higher infection rates within households of infected individuals. In contrast, the detection rates of strategies B1 and B2 were expected to differ because of the different effects of the upstream prescreening mechanisms.

At the time of planning the study, the 4-week cumulative SARS-CoV-2 prevalence was estimated to be 0.25% in the region, based on data from the first surge [50], mainly sourced from passive surveillance (ie, symptomatic persons). Assuming a similarly high proportion of asymptomatic SARS-CoV-2 carriers, the overall prevalence was estimated to be approximately 0.5%. Thus, in the most representative study arm A1, which used a purely random sample to directly reflect the pandemic events, a total of 2500 gargle samples could estimate such a prevalence with a relative precision of −0.35% to +0.35% (95% CI) and a power of 95% [51]. Hence, assuming a 50% response rate after one-time prompting, at least 5000 addresses were required for strategy A1. For strategy A2, assuming an average household size of 2 and a response rate of 50% after prompting, a gross sample of at least 2500 addresses was necessary [52]. For strategy B1, we assumed an 80% response to the sampling request after positive prescreening; hence, at least 3125 sample kits had to be mailed out to yield 2500 tested samples. On the basis of this and assuming a SARS-CoV-2 prevalence of 0.5% and a prescreening tool sensitivity of 90% and specificity of 70%, at least 10,313 participants had to complete the initial prescreening. Furthermore, expecting a 50% response to the prescreening request and 50% response to prompting required an initial gross sample of at least 13,750 addresses. Similarly, for strategy B2, a gross sample of at least 6875 addresses was required (average household size of 2). Consequently, a total gross sample of 28,125 addresses was required, and the participants were allocated in a ratio of 10:5:28:14 to the study arms (Figure S1 in Multimedia Appendix 2) [39].

### Randomization

A stratified general population representative sample with 2 strata (Rhine-Neckar district plus Heidelberg city) and 2 different sampling approaches (Heidelberg—simple random sample and Rhine-Neckar—2 stage cluster sampling) were used to draw 3 weekly batches to avoid negative impacts on the response rate owing to uncertain external circumstances (such as holidays or a higher rate of infections). Because of the considerably larger population of Heidelberg city, the stratification was used to avert a decrease in statistical power for estimation in rural areas. The batches were then divided among 15 consecutive recruitment weekdays and randomly assigned to the 4 study arms. The gross sample included 21,739 individuals in the Rhine-Neckar district and 6386 individuals in the Heidelberg stratum. Potential participants were randomly drawn from municipalities’ (51/53, 96%) population registers as samples proportional to the population size of the municipalities (Multimedia Appendix 2) [39].

The 2-stage sampling was designed by the Leibnitz Institute for Social Sciences in Mannheim, Germany. The Heidelberg Institute of Global Health requested the addresses from the municipalities.

### Recruitment and Study Materials

Study recruitment started on November 18, 2020, and ended on December 11, 2020, and one-time reminders were sent until December 16, 2020. The general implementation (including testing activities and hotlines) lasted until December 23, 2020.

The study website displayed information in 5 languages (German, Turkish, English, Russian, and Italian) and contained a gargling demonstration video. The study information (brochure and website) and the consent forms were separately presented in adequate language for children (aged 7-11 years), adolescents (aged 12-17 years), parents, and adults. In addition, a hotline was implemented and made accessible during the study period (weekdays 7-11 AM and 2-6 PM).

A prescreening questionnaire was provided to all initially contacted individuals. Its completion was voluntary in A1 and A2 and mandatory in B1 and B2 study arms.

### Logistics

The study arms A1 and A2 immediately received gargle sample collection kits, whereas the study arms B1 and B2 had to complete the prescreening questionnaire first. The differences in logistics in are discussed in detail as follows:

1. Study arm A1: Randomly selected individuals (hereinafter referred to as initially contacted individuals) received an invitation letter by post containing the study information, a sample collection kit, and a stamped biohazard return envelope (Multimedia Appendix 3).
2. Study arm A2: The procedures were the same as in A1 with the difference that the initially contacted individuals received 4 kits to sample household members. Additional kits could be ordered by email or via the hotline if needed.
3. Study arm B1: Initially contacted individuals first received an invitation letter with the study information, a stamped return envelope, and the prescreening questionnaire, asking about 16 typical COVID-19 symptoms to be completed web-based or in print. Next, the questionnaire was evaluated using a random forest algorithm that had been trained on data sets of patients with COVID-19 to classify participants into COVID-19–free and potentially sick individuals. If the questionnaire was flagged by the algorithm, the individual received a second envelope containing the sample collection kit and a stamped biohazard return envelope.
4. Study arm B2: The procedures were the same as in B1 with the difference that the initially contacted individuals who were classified as having COVID-19 by the prescreening algorithm subsequently received 4 sample collection kits for household members.

In each study arm, participants who received the sample collection kits were asked to collect a gargle sample themselves after gargling 5 mL of saline at home and return it to the laboratory (Multimedia Appendices 2 and 3). The participants were expected to take the sample latest 1 day after they received the sample collection kit.

### Laboratory and Blinding

To enable scale-up of testing while maintaining sensitivity, an RT-LAMP was used for analyzing the liquid gargle samples. Weakly (<3 replicates positive) and clearly positive gargle samples (all replicates positive) were subsequently analyzed using RT-PCR as a confirmation test [24-26].

Laboratory staff who conducted the RT-LAMP and the RT-PCR confirmation tests were blinded with regard to the study arms. The vials carried a unique barcode only, and the laboratory data were kept on a separate server; linkage of laboratory data with participants’ data was performed after the laboratory analysis was completed.

### Data Processing

Data cleaning was performed using SAS software (version 9.4 TS1M4; SAS Institute Inc). Statistical analyses were performed using R (version 4.1.1; R Foundation for Statistical Computing) and Stata (version 15.1; StataCorp).

### Statistical Methods

#### Prescreening Questionnaire

We developed a symptom screening algorithm using machine learning. The underlying data sets were from various settings with and without SARS-CoV-2 infected patients, including samples from a general population screen and persons tested for SARS-CoV-2 infection because of symptoms or high-risk exposure (Multimedia Appendix 2). The threshold of the algorithm was optimized toward sensitivity (80% sensitivity and 75% specificity).

#### Descriptive Statistics

Differences in demographics, frequency of COVID-19 symptoms, and epidemiological variables between the trial arms were assessed using the chi-square test and ANOVA (no adjustment for multiple testing). The significance level α was set at 5%.

#### Prevalence Estimation

The total number of SARS-CoV-2 cases was calculated using the Horvitz-Thomson estimator to account for unequal selection probabilities in the 2-stage sampling [40]. The individual probability to get selected for participation was based on the number of primary sampling units per municipality, the population in this municipality obtained from the Leibniz-Institute for the Social Sciences, and was normalized for each study arm separately to match the number of performed tests. The Brewer approximation was used to calculate the estimate’s variance [41]. We refrained from calculating cluster-adjusted prevalence in A2 and B2 as the number of positive cases was too small to take the within household transmission rates into account. To compare the results of our study with the reported number of cases, we estimated the reported prevalence based on official data from the Robert Koch Institute (RKI; Multimedia Appendix 2).

#### Sensitivity Analysis

The trial was implemented in parallel to the existing passive surveillance system; hence, persons could have been captured by both systems. Some hotline callers refused to participate because they had already tested positive in the passive surveillance. Therefore, we conducted a sensitivity analysis and recalculated the prevalence estimates to account for missed positive cases owing to overlap with passive surveillance (Multimedia Appendix 2).

## Results

### Recruitment

**Figure 1:**
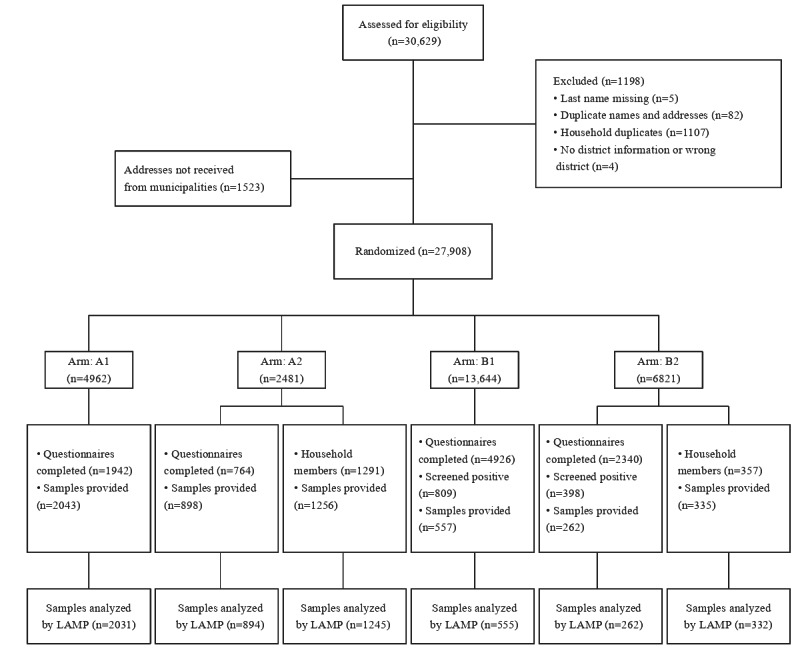
Study flowchart. A1: individuals without mandatory prescreening; A2: households without mandatory prescreening; B1: individuals with mandatory prescreening; B2: households with mandatory prescreening; LAMP: loop-mediated isothermal amplification.

**Figure 2:**
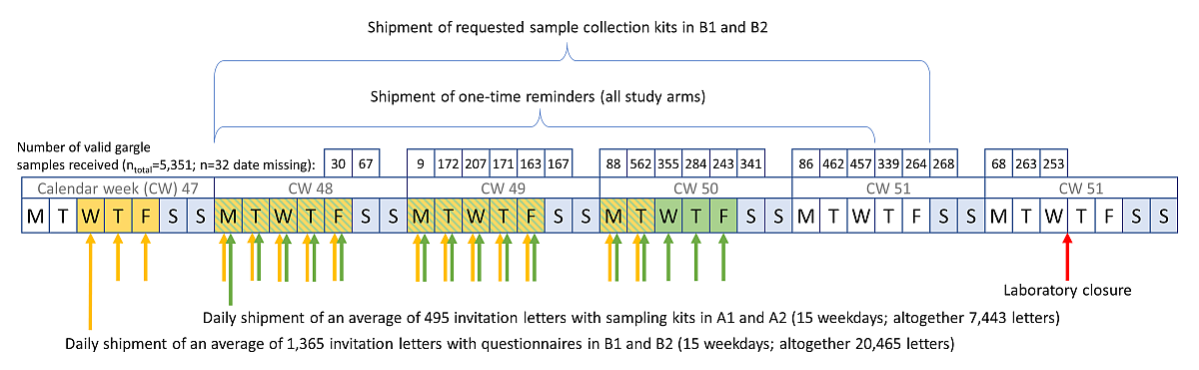
Study timeline. A1: individuals without mandatory prescreening; A2: households without mandatory prescreening; B1: individuals with mandatory prescreening; B2: households with mandatory prescreening.

Altogether, 30,629 addresses were provided by 51 municipalities’ registration offices. After excluding duplicates, 27,908 (99.23%) addresses out of the planned sample size of 28,125 were randomly allocated to the arms (Figure 1; Table S1 in Multimedia Appendix 2). Considering the longer individual study course in B1 and B2 because of the prescreening questionnaire, shipment of study materials and recruitment of participants started on November 18, 2020, for B1 and B2 and on November 23, 2020, for A1 and A2 and stopped on December 8, 2020, for B1 and B2 and on December 11, 2020, for A1 and A2 (Figure 2). Similar gargle sampling days should be achieved for all study arms. In total, 11,855 participants were recruited.

### Demographics

The participants were slightly older than the general population (mean age 44.5 years in 2019; [53]), mainly because of the exclusion of children aged <7 years (Table S2 in Multimedia Appendix 2; Table 1). The study arms differed with regard to the education level of participants who provided a valid questionnaire (Table 2).

**Table 1:** Demographics of initially contacted individuals who decided to participate (initially contacted individuals who either provided a sample [A1 and A2] or a questionnaire [B1 and B2]).

Demographics		A1^a^ (n=2043)	A2^b^ (n=898)	B1^c^ (n=4926)	B2^d^ (n=2340)	Total (n=10,207)	*P* value
**Sex^e^, n (%)**							.64^f^
	Female	1066 (52.18)	471 (52.45)	2615 (53.09)	1265 (54.06)	5417 (53.07)	
	Male	977 (47.82)	426 (47.44)	2311 (46.91)	1075 (45.94)	4789 (46.92)	
	Missing^g^	0 (0)	1 (0.11)	0 (0)	0 (0)	1 (0.01)	
**Age (years)^e^**							.52^h^
	Value, mean (SD; range)	46.8 (20.4; 7-99)	47.5 (20.6; 7-93)	47.2 (20.7; 7-98)	47.7 (20.6; 7-98)	47.3 (20.6; 7-99)	
	Missing, n (%)^g^	0 (0)	1 (0.11)	0 (0)	27 (1.15)	28 (0.27)	

^a^A1: individual participants without prescreening.

^b^A2: initially contacted individuals in households who decided to participate without prescreening.

^c^B1: individual participants with prescreening.

^d^B2: initially contacted individuals in households with prescreening.

^e^Age and sex were provided by the registration offices.

^f^Pearson chi-square test.

^g^Number of participants with missing information.

^h^Linear model ANOVA.

**Table 2:** Demographics of participants who provided a valid questionnaire.

Demographics		A1^a^ (n=1942)	A2^b,c^ (n=824)	B1^d^ (n=4926)	B2^e,f^ (n=2346)	Total (n=10,038)	*P* value
**Sex^g^, n (%)**							.68^h^
	Female	1019 (52.47)	427 (52.07)	2615 (53.09)	1267 (54.05)	5328 (53.11)	
	Male	923 (47.53)	393 (47.93)	2311 (46.91)	1077 (45.95)	4704 (46.89)	
	Missing^i^	0 (0)	4 (0.49)	0 (0)	2 (0.08)	6 (0.06)	
**Age (years)^g^**							.33^j^
	Values, mean (SD; range)	46.7 (20.5; 7-99)	46.6 (20.7; 7-93)	47.2 (20.7; 7-98)	47.7 (20.5; 7-98)	47.2 (20.6; 7-99)	
	Missing, n (%)^i^	0 (0)	6 (0.73)	0 (0)	2 (0.09)	8 (0.08)	
**Education, n (%)**							<.001^h^
	Doctor of Philosophy	121 (6.97)	49 (6.55)	286 (6.29)	150 (6.94)	606 (6.59)	
	Master studies or diploma studies	416 (23.95)	182 (24.33)	1041 (22.9)	538 (24.9)	2177 (23.68)	
	Bachelor studies	171 (9.84)	60 (8.02)	393 (8.64)	177 (8.19)	801 (8.71)	
	Master craftsman training	99 (5.7)	39 (5.21)	236 (5.19)	99 (4.58)	473 (4.15)	
	High school or technical or economic high school	267 (15.37)	144 (19.25)	775 (17.05)	327 (15.13)	1513 (16.46)	
	Vocational school	236 (13.59)	97 (12.97)	541 (11.9)	244 (11.29)	1118 (12.16)	
	Secondary school	330 (19.0)	126 (16.84)	1072 (23.58)	532 (24.62)	2060 (22.41)	
	No degree or still in school education	97 (5.58)	51 (6.82)	202 (4.44)	94 (4.35)	444 (4.83)	
	Missing^i^	205 (10.56)	76 (9.22)	380 (7.71)	185 (7.89)	846 (8.43)	
**Job description, n (%)**							.04^h^
	Managers	78 (4.72)	26 (3.61)	215 (5.14)	108 (5.38)	427 (4.99)	
	Professionals	428 (25.92)	174 (24.13)	907 (21.68)	478 (23.83)	1987 (23.21)	
	Technicians and associate professionals	169 (10.24)	84 (11.65)	467 (11.16)	232 (11.57)	952 (11.12)	
	Clerical support workers	90 (5.45)	36 (4.99)	230 (5.5)	89 (4.44)	445 (5.2)	
	Services and sales workers	135 (8.18)	48 (6.66)	289 (6.91)	130 (6.48)	602 (7.03)	
	Skilled agricultural, forestry, and fishery workers	3 (0.18)	6 (0.83)	15 (0.36)	7 (0.35)	31 (0.36)	
	Craft and related trades workers	65 (3.94)	31 (4.3)	156 (3.73)	71 (3.54)	323 (3.77)	
	Plant and machine operators and assemblers	15 (0.91)	2 (0.28)	40 (0.96)	31 (1.55)	88 (1.03)	
	Elementary occupations	29 (1.76)	15 (2.08)	63 (1.51)	37 (1.84)	144 (1.68)	
	Not part of the labor force	577 (34.95)	267 (37.03)	1614 (38.58)	746 (37.19)	3204 (37.42)	
	Other	9 (0.55)	5 (0.69)	27 (0.65)	10 (0.5)	51 (0.6)	
	Ambiguous or incomplete responses	53 (3.21)	27 (3.74)	161 (3.85)	67 (3.34)	308 (3.6)	
	Missing^i^	291 (14.98)	103 (12.5)	742 (15.06)	340 (14.49)	1476 (14.70)	
**Employment status, n (%)**							.26^h^
	Employed but not specified	849 (51.52)	356 (49.44)	1973 (47.26)	987 (49.28)	4165 (48.74)	
	Self-employed paid work	23 (1.4)	7 (0.97)	76 (1.82)	35 (1.75)	141 (1.65)	
	Nonself-employed paid work	122 (7.4)	46 (6.39)	302 (7.23)	156 (7.79)	626 (7.33)	
	Service contract	1 (0.06)	0 (0)	2 (0.05)	0 (0)	3 (0.04)	
	Incapacitated for work	2 (0.12)	0 (0)	6 (0.14)	3 (0.15)	11 (0.13)	
	Working from home	5 (0.3)	1 (0.14)	16 (0.38)	4 (0.2)	26 (0.3)	
	Parental leave	13 (0.79)	14 (1.94)	39 (0.93)	22 (1.1)	88 (1.03)	
	Unpaid family workers	10 (0.61)	4 (0.56)	19 (0.46)	9 (0.45)	42 (0.49)	
	Pupil	47 (2.85)	26 (3.61)	155 (3.71)	59 (2.95)	287 (3.36)	
	Student	108 (6.55)	45 (6.25)	289 (6.92)	121 (6.04)	563 (6.59)	
	Internship	3 (0.18)	0 (0)	5 (0.12)	3 (0.15)	11 (0.13)	
	Further education (full time, long term)	1 (0.06)	2 (0.28)	3 (0.07)	1 (0.05)	7 (0.08)	
	Doctor of Philosophy or doctorate	9 (0.55)	3 (0.42)	23 (0.55)	8 (0.4)	43 (0.5)	
	Traineeship in a school or law firm	1 (0.06)	2 (0.28)	6 (0.14)	4 (0.2)	13 (0.15)	
	Vocational training	16 (0.97)	12 (1.67)	76 (1.82)	26 (1.3)	130 (1.52)	
	Pensioners	38 (2.31)	19 (2.64)	77 (1.84)	34 (1.7)	168 (1.97)	
	Retirement	328 (19.9)	158 (21.94)	881 (21.1)	412 (20.57)	1779 (20.82)	
	Partial retirement	12 (0.73)	2 (0.28)	20 (0.48)	13 (0.65)	47 (0.55)	
	Unemployed	49 (2.97)	16 (2.22)	179 (4.29)	93 (4.64)	337 (3.94)	
	Other (eg, military, civilian service, holiday, or illness)	4 (0.24)	3 (0.42)	10 (0.24)	2 (0.1)	19 (0.22)	
	Undetermined	7 (0.42)	4 (0.56)	18 (0.43)	11 (0.55)	40 (0.47)	
	Missing^i^	294 (15.14)	104 (12.62)	751 (15.24)	343 (14.62)	1492 (14.86)	
Household size, mean (SD)		2.64 (1.29)	2.73 (1.24)	2.66 (1.38)	2.65 (1.43)	2.66 (1.39)	.04^k^

^a^A1: individual participants without mandatory prescreening who voluntarily completed the prescreening questionnaire.

^b^A2: household members without mandatory prescreening who voluntarily completed the prescreening questionnaire.

^c^n=764 initially contacted individuals (n=730+34 initially contacted individuals who did not provide a valid sample or consent but answered or consented the questionnaire) plus 60 household members who additionally filled out a questionnaire.

^d^B1: individual participants with mandatory prescreening.

^e^B2: initially contacted individuals in households with mandatory prescreening.

^f^n=2340 initially contacted individuals plus 6 household members who additionally filled out a questionnaire.

^g^Age and sex were available for initially contacted individuals from the registration offices and for household members from the consent form.

^h^Pearson chi-square test.

^i^Number of participants with missing information.

^j^Linear model ANOVA.

^k^Welch 2-sample *t* test (2-tailed) between study arms A2 and B2.

### Response Rates

**Figure 3:**
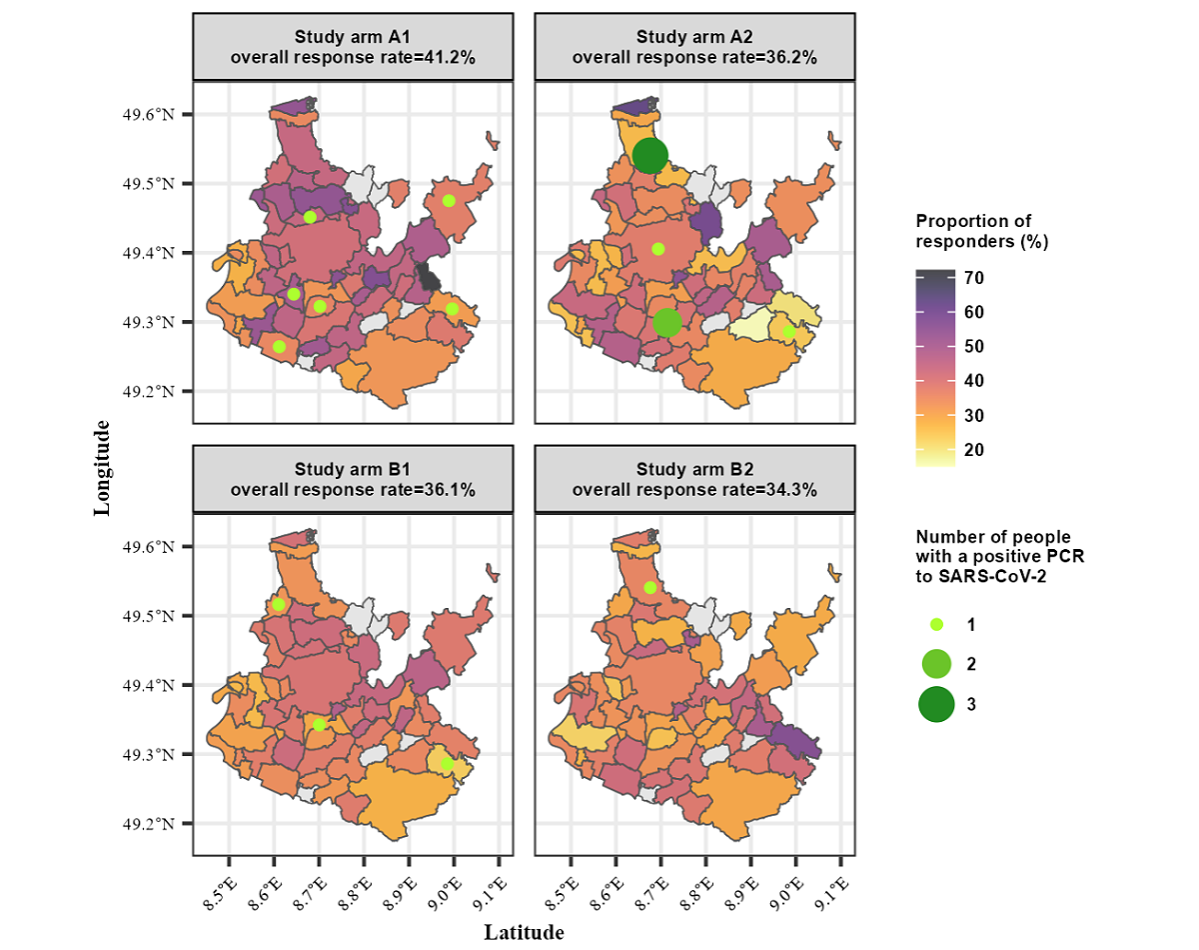
Response rates in the 51 municipalities; newly detected SARS-CoV-2 infections by study arm (the gray areas indicate municipalities randomly excluded in the cluster sampling approach). A1: individuals without mandatory prescreening; A2: households without mandatory prescreening; B1: individuals with mandatory prescreening; B2: households with mandatory prescreening; PCR: polymerase chain reaction.

Overall, 36.57% (10,207/27,908) of the individuals who were contacted responded by either directly providing a gargle sample (A1 and A2) or by completing the prescreening questionnaire (B1 and B2; Table 3). The response rates (A1: 41.2%, A2: 36.2%, B1: 36.1%, and B2: 34.3%) differed significantly among the study arms (*P*<.001; chi-square test), with the study arms involving household members responding the least. The lowest variation in response rates across the study region could be observed in A1 and B1 (Figure 3). Nonresponders were younger (44.2 years vs 46.6 years; *P*<.001) and comprised a larger proportion of males (50.5% vs 47.3%; *P*<.001). However, there were no additional study arm–related selection effects (responders’ age: ANOVA *P*=.52; sex: chi-square *P*=.64; Table 1).

**Table 3:** Response rates and test results by study arm.

		No prescreening questionnaire		Prescreening questionnaire		Total
		Arm A1^a^	Arm A2^b^	Arm B1^c^	Arm B2^d^	
People contacted/group, n		4962	2481	13,644	6821	27,908
**Participation rate**						
	Number of responders/total number of people contacted, n/N (%)^e^	2043/4962 (41.17)	898/2481 (36.19)	4926/13,644 (36.10)	2340/6821 (34.31)	10,207/27,908 (36.57)
Household members, n		N/A^f^	1291	N/A	357	1648
**Questionnaires completed by initially contacted individuals**						
	Number completed/total number of responders, n/N (%)	1894/2043 (92.71)^g^	730/898 (81.29)^g^	4926/4926 (100)	2340/2340 (100)	9890/10,207 (96.89)
**Initially contacted individuals classified as having COVID-19 in the prescreening**						
	Number classified as having COVID-19/total number of responders, n/N (%)	N/A	N/A	809/4926 (16.42)	398/2340 (17.01)	1207/7266 (16.61)
**Samples provided by initially contacted individuals**						
	Number of samples provided/number of responders or classified as having COVID-19 in the prescreening, n/N (%)	2043/2043 (100)	898/898 (100)	557/809 (68.85)	262/398 (65.83)	3760/4148 (90.65)
**Samples provided by household members**						
	Number of samples/total number of participating household members, n/N (%)	N/A	1256/1291 (97.29)	N/A	335/357 (93.84)	1591/1648 (96.54)
**Samples tested for SARS-CoV-2 by LAMP^h^ assay**						
	Number of samples tested/total number of samples received, n/N (%)	2031/2043 (99.41)	2139/2154 (99.30)^i^	555/557 (99.64)	594/597^j^ (99.5)	5319/5351 (99.40)
**Samples with (weakly) positive result by the LAMP assay^k^**						
	Number of samples with (weakly) positive LAMP results/number of samples tested, n/N (%)	26/2031 (1.28)	30/2139 (1.40)	9/555 (1.62)	10/594 (1.68)	75/5319 (1.41)
**Samples with a positive result by the LAMP assay^l^**						
	Number of samples with positive LAMP result/number of samples with (weakly) positive LAMP results, n/N (%)	5/26 (19.23)	5/30 (16.67)	3/9 (33.33)	0/10 (0)	13/75 (17.33)
Total positive cases confirmed by PCR^m^, n		6	7	3	1	17
**Thereof weakly positive LAMP results confirmed positive by PCR^n^**						
	Number of positive PCR results/number of samples with (weakly) positive LAMP results, n/N (%)	1/26 (3.85)	2/30 (6.67)	0/9 (0)	1/10 (10)^o^	4/75 (5.33)
**Thereof LAMP positive samples confirmed positive by PCR^p^**						
	Number of positive PCR results/number of samples with positive LAMP result, n/N (%)	5/5 (100)	5/5 (100)^q^	3/3 (100)	0/0 (0)	13/13 (100)
**Asymptomatic cases^r^**						
	Number of asymptomatic cases/number of cases with prescreening data, n/N (%)	2/6 (33.33)	1/2 (50)	0/3 (0)	0/0 (0)	3/17 (17.65)

^a^A1: individuals without mandatory prescreening.

^b^A2: households without mandatory prescreening.

^c^B1: individuals with mandatory prescreening.

^d^B2: households with mandatory prescreening.

^e^Defined as the number of people initially contacted who provided a gargle liquid sample (arms A1 and A2) or who answered the prescreening questionnaire (arms B1 and B2), referred to as “responders,” out of the total number of people contacted in each arm.

^f^N/A: not applicable.

^g^7 questionnaires in A1 and A2 could not be considered in the random forest algorithm because of missing values in the paper version.

^h^LAMP: loop-mediated isothermal amplification.

^i^The denominator consists of the number of samples provided by initially contacted individuals in study arm A2 and their household members. The numerator consists of the samples tested by LAMP for initially contacted individuals (n=898) plus the samples tested by LAMP for household members (n=1241).

^j^The denominator consists of the number of samples provided by initially contacted individuals in study arm B2 and their household members. The numerator consists of samples tested by LAMP for initially contacted individuals (n=262) plus the samples tested by LAMP for household members (n=332).

^k^Samples with an initial weakly positive SARS-CoV-2 result by the LAMP assay.

^l^Samples that initially had a (weakly) positive SARS-CoV-2 result by the LAMP assay and that tested LAMP positive for SARS-CoV-2 upon retesting.

^m^PCR: polymerase chain reaction.

^n^Samples that initially had a weakly positive SARS-CoV-2 result by the LAMP assay, followed by a PCR positive test without an intermediate LAMP positive test.

^o^Out of 1 PCR positive sample, 1 is from a household member.

^p^Samples that tested LAMP positive for SARS-CoV-2 and were then confirmed positive by PCR.

^q^Of the 5 PCR positives for SARS-CoV-2, a total of 3 were from household members.

^r^Cases without any of the following symptoms among all positive cases: fever, cough, cough with sputum, sore throat, difficulty breathing, exhaustion, headache, runny nose, muscle ache, chest pain, diarrhea, nausea, no taste or smell, chills, short breath, and confused; denominator: total number of PCR samples with questionnaire data available.

### Outcomes

Although the prescreening was not compulsory in A1 and A2, approximately 92.71% (1894/2043) of the participants in A1 and 81.3% (730/898) of the initially contacted individuals in A2 completed the optional questionnaire. The random forest algorithm classified approximately 16.61% (1207/7266) of the initially contacted individuals in B1 and B2 to have suspicious COVID-19 symptoms (Table 3). Of those who were classified as having COVID-19 in the prescreening, only 68.9% (557/809) provided a valid gargle sample upon subsequent request in B1 and 65.8% (262/398) provided a valid gargle sample upon subsequent request in B2. However, of the household members, 97.29% (1256/1291) provided valid gargle samples in A2 and 93.8% (335/357) provided valid gargle samples in B2.

In all study arms, 99.40% (5319/5351) of the gargle samples were analyzed; only 0.60% (32/5351) of the samples could not be processed because of the viscosity being too high. All positive LAMP results were confirmed by RT-PCR. Of the weakly positive LAMP results, approximately 5% yielded a positive result in the PCR analysis. Overall, 17 SARS-CoV-2 infections were detected in this study (Figure 3; Table 3).

Of the 17 positive cases, 8 (47%) were symptomatic and 3 (18%; n=2, 67% adults in A1 and n=1, 33% child in A2) were completely asymptomatic SARS-CoV-2 carriers. Of the 8 symptomatic cases, 6 (75%) had <3 acute COVID-19 symptoms (n=4, 67% A1 and A2 and n=2, 33% B2), 1 (13%) had 8 symptoms (A1), and 1 (13%) had all 16 symptoms (B2). COVID-19 symptom data were missing for 5 participants in A2 (voluntary questionnaire) and 1 participant in B2 (household member); hence, some asymptomatic cases may not have been counted.

Overall, 8 hotline callers reported having recently tested positive and declined to provide a gargle sample. Of the 8 hotline callers, 5 (63%) received the sample collection kit or called the hotline within 14 days of testing positive elsewhere. These cases might have been detected in our study as well. In addition, as recorded at the local health authority, 51 of the nonresponders had been tested positive elsewhere within 14 days before our invitation (not considered in the sensitivity analysis).

**Figure 4:**
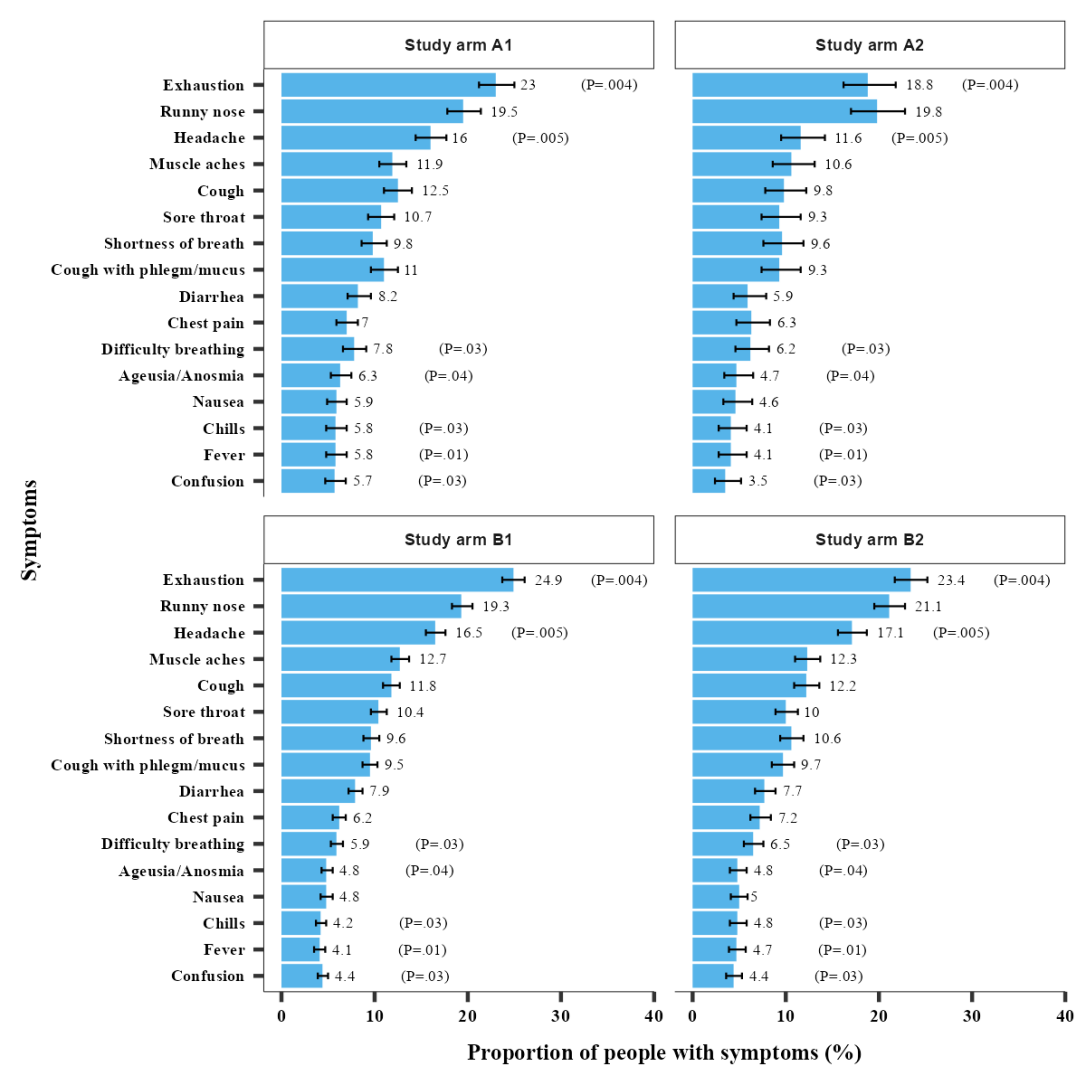
Frequency of COVID-19–related symptoms among those who filled out the prescreening questionnaire (initially contacted individuals only; *P* values based on Pearson chi-square test). A1: individuals without mandatory prescreening; A2: households without mandatory prescreening; B1: individuals with mandatory prescreening; B2: households with mandatory prescreening.

The arms differed substantially regarding some COVID-19 symptoms (Figure 4; Table S3 and Figures S2 and S3 in Multimedia Appendix 2). Approximately 20.60% (2054/9972) either worked with children or in the medical field (Table S4 in Multimedia Appendix 2).

### Prevalence Estimates

**Figure 5:**
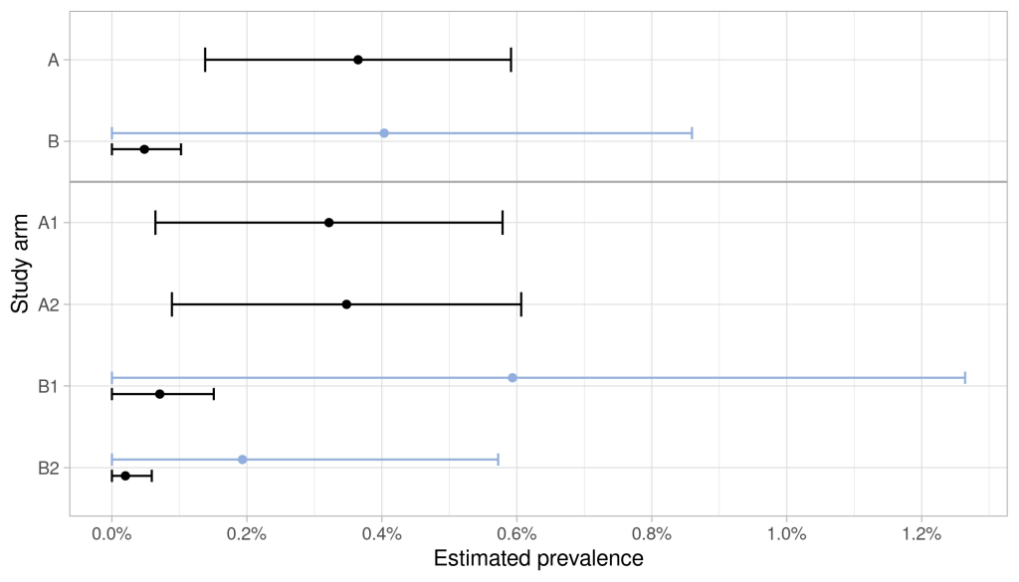
Weighted prevalence estimates in study arms A1 (individuals without mandatory prescreening) and A2 (households without mandatory prescreening) combined, B1 (individuals with mandatory prescreening) and B2 (households with mandatory prescreening) combined, and each arm separately. Light blue indicates the fraction of positive cases among all the performed tests. For combined arms, only the initially contacted participants are included to avoid household bias in arms A2 and B2.

**Figure 6:**
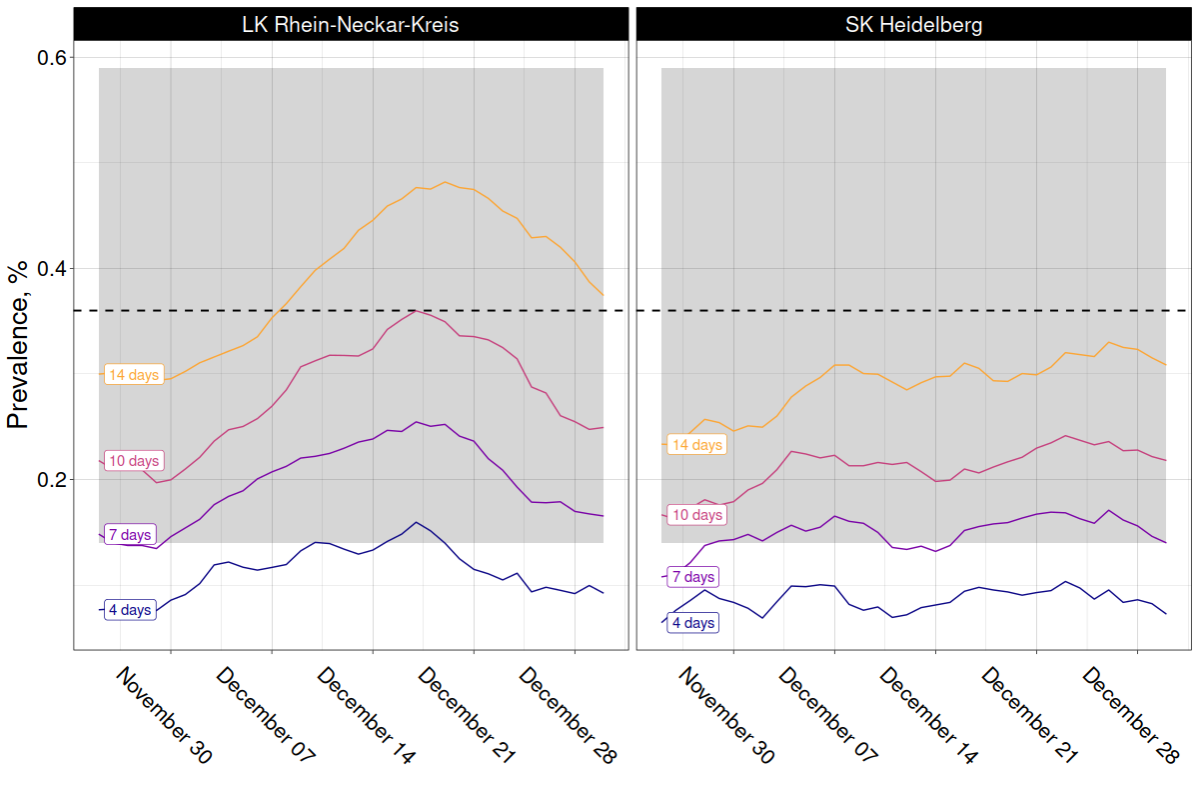
Variation in the prevalence estimate derived from Robert Koch Institute incidence data, depending on the assumed duration of polymerase chain reaction positivity (each prevalence curve is labeled by the corresponding duration in days; horizontal dashed line is the estimated prevalence based on the combined results from arms A1 and A2, with 95% CI indicated by the shaded area).

The weighted prevalence estimates (initial cases only) differed considerably between combined arms A with 0.36% (95% CI 0.14%-0.59%) and B with 0.05% (95% CI 0.00%-0.10%). However, we did not detect a significant difference between the single arms (household members in A2 and B2 included). In A1, the prevalence was 0.32% (95% CI 0.06%-0.58%), and in A2, the prevalence was 0.35% (95% CI 0.09%-0.61%), with lower estimates in B1 and B2 (0.07%, 95% CI 0.00%-0.15% and 0.02%, 95% CI 0.00%-0.06%; Figure 5; Table S5 in Multimedia Appendix 2). We also compared our daily case estimates with the RKI data (Figure S4 in Multimedia Appendix 2). Assuming that the participants remained positive on average 14 days, the estimated prevalence range based on RKI data (0.23%-0.48%) stayed within the 95% CI 0.14-0.59 of our estimate for the combined arms A. Assuming an average duration of 10 days, the RKI-based prevalence range (0.16%-0.36%) was completely within the lower half of our 95% CI (Figure 6). Figure S5 in Multimedia Appendix 2 depicts the minimum number of positive cases that should have been detected in the trial, with the lower bound determined by the prevalence estimate based on RKI data (Figure 6). If we additionally considered the hotline callers that tested positive elsewhere, the prevalence increased further to 0.37% (95% CI 0.10%-0.65%) in A1, 0.35% (95% CI 0.09%-0.61%) in A2, 0.14% (95% CI 0.03%-0.26%) in B1, and 0.07% (95% CI 0.00%-0.15%) in B2 (Figure S6 and Table S5 in Multimedia Appendix 2).

## Discussion

### Principal Findings

To the best of our knowledge, this is the only study to date that has simultaneously tested different approaches to active SARS-CoV-2 surveillance for the general public based on daily renewed random and population representative samples in a seamless chronology. In this paper, we focused on the feasibility and epidemiological outcomes of the trial.

Our study demonstrated that in general, the combination of postal mailing of sample collection kits and return of gargle liquid samples followed by high-sensitivity RT-LAMP analysis is a feasible way to conduct active SARS-CoV-2 surveillance in the general population without placing an additional burden on the capacity of PCR analysis and routine diagnostic testing [26]. Furthermore, our study suggests that the primarily symptom-based prescreening did not add benefit to case finding but rather complicated processes. Even if optimized, such a tool is unlikely to capture presymptomatic or asymptomatic cases and might have to be adapted with each new SARS-CoV-2 variant. The surveillance strategies could detect additional cases, some of them being asymptomatic, which were not captured by passive surveillance.

We were able to design and implement this complex trial in just 8 weeks by using innovative digital tools, agile development, and flat communication structures, which resulted in highly digitized processes and ensured that the study logistics were highly functional from the program outset. We are, therefore, convinced that a similar active pandemic surveillance system deployed outside a scientific study could be set up even faster, provided appropriate laboratory infrastructure and open-source software are held at the ready and standards are introduced [54].

Active SARS-CoV-2 surveillance should capture a sufficient proportion of symptomatic and asymptomatic cases to allow continuous estimation of the true prevalence trend with sufficient precision. However, despite the random drawing of potential participants, the nonresponders in our study were not missing at random, as selection owing to voluntary self-enrollment occurred, which was reflected in the younger and more male responders on average. Taking this into account, study arm A1 (random selection of individuals) was most likely to yield the best estimate of the true prevalence, although it was slightly biased because of voluntary self-enrollment. Investing in measures (eg, incentives and analytical corrections) to minimize the proportion of nonresponders may further reduce bias and increase accuracy.

The prevalence estimates derived from the RKI data based on passive surveillance constituted the lower bound of the prevalence estimates. As expected, the prevalence detected in our study tended to be higher, and the estimates of A1 and A2 were the most accurate. However, we cannot make a definitive statement regarding the most effective strategy because the number of positive cases was too low because of the actual sample size and the stage of the pandemic. It is important to note that this study was a research project not embedded in the public health system with data fusion and reconciliation. The inclusion of theoretically detectable cases who refused to participate but reported a recent infection to the study hotline upwardly adjusted the prevalence estimates. If other nonresponders who tested positive shortly before the invitation had been considered, the prevalence estimate would have been well above the official figures.

### Study Participation

The overall participation rate of 36.57% (10,207/27,908) was lower than the envisaged 50%. First, the complexity of the information material necessary for a 4-arm trial might have been difficult to understand, thereby hindering a timely and appropriate participation [55]. Second, the different demands on the participants in a difficult overall situation (ie, lockdown) and an increasing pandemic fatigue before Christmas 2020 might have influenced the response [55]. One complicating aspect, the requested sampling before breakfast, seems to be less relevant based on new data (Multimedia Appendix 2).

Complementary qualitative data from approximately 80 responders and nonresponders shed light on these barriers. For example, some of the nonresponders thought they were selected because they participated in other studies or because acquaintances had to quarantine themselves. The study invitation also triggered fear of being infected in a few selected persons. The amount of information and perceived test complexity overwhelmed many [55].

Our study participation rates were similar to or exceeded those of other studies. In the United Kingdom, repeated community-based RT-PCR testing based on self-administered throat and nasal swabs estimated prevalence on a monthly basis. The number of invitation letters sent between May 2020 and December 2021 increased from 395,000 to 804,000, but the response rate (number of tests/letters) decreased from 30.5% to 12.1% and the PCR test return rate (number of tests performed/kits requested) varied between 67.2% and 78.9% [56]. During the same period as our study, 28.4% of the invitees requested sample collection kits, and the actual response rate was 22.4% [57]. We provided sample collection kits immediately along with the invitation letter in A1 and A2. In addition, we requested a gargle sample instead of a swab sample, which resulted in response rates of 41.2% and 36.2%, respectively. This may indicate that a low-barrier approach and an alternative to throat and nasal swabs could unlock further potential for participation.

Some methodological changes could have increased participation. Government support, media campaigns, and adding incentives could foster participation [58,59]. This might be particularly important if rapid antigen tests are widely available; therefore, adequate but not overly complicated information should educate participants about test quality and the purpose of active surveillance. Furthermore, the methodology was tailored to the ethical requirements for scientific trials, the mere extent of information materials might have deterred, and the label of a scientific study might have unsettled potential participants [55]. In contrast, routine implementation accompanied by a legal framework would only have to follow the general data protection laws, simplifying paperwork, and could be integrated into pandemic containment strategies, eliminating the chance for double sampling by linking data with passive surveillance systems.

### Acceptability of Self-Sampling

In our study, mail-in gargle self-sampling proved to be feasible. The method was perceived as pleasant, allowed for high test accuracy in asymptomatic patients, and reduced the risk of spreading infections. Liquid gargle sampling was valued as more convenient than nasopharyngeal swabs, and although self-sampling was cumbersome for some older participants, many respondents found accurate self-sampling with gargling easier. The acceptability was mainly driven by communication (content of information, letters, support system, and rapidity of test results) and trust in the sampling or laboratory-based testing method [55]. Gargling a saline solution is a low-barrier sampling method applicable in almost all age groups [60] and suitable not only for LAMP but also for PCR analysis. Independently, the RT-LAMP analysis allows high-throughput testing with low costs and can be combined with nasopharyngeal, nasal, or throat swab samples without compromising sensitivity. Sample pooling can further multiply the testing capacity [21,22].

### Cost-Effectiveness

From an epidemiological perspective, we identified A2 as the most cost-effective strategy, closely followed by A1, based on evidence from a parallel economic evaluation [37]. We postulate that surveillance strategies similar to A1 and A2 likely yield the best estimates and are most efficient. They are also logistically simpler than the strategies with a prescreening tool.

### Limitations

This study had several limitations. First, the actual execution of the trial was limited to 3 weeks; time constraints limited adequate testing of the prescreening tool and impeded pilot studies. Second, random samples from most municipalities yielded a representative pool of potential participants; however, Heidelberg and its surrounding area have a higher socioeconomic level than an average German municipality. Voluntary participation likely correlates with gradients in wealth; thus, the participation rate could have been different elsewhere in Germany. As high socioeconomic strata are simultaneously less susceptible to SARS-CoV-2 infection and more likely to participate in studies, this possibly led to slightly underestimated prevalence estimates [61]. Third, participants in each study arm were expected to return samples no later than 1 day after receiving the sample collection kits. However, for some participants, the instructions in the invitation letter were not clear enough as this could not be tested in a pilot study owing to time constraints. Therefore, some participants collected samples on a day that was more convenient for them [55]. However, these untimely samples were most likely random, which may explain the lower sample return at the beginning and some late samples. Clear communication could yield timely sample return and thus more stable return numbers throughout the surveillance period. Finally, the calculation of the sample size and thus the accuracy of the prevalence were limited by the available financial resources and various uncertainties in the assumptions.

We expected to receive a similar number of gargle samples in each arm to enable systematic bias estimation. Because B1 uses a symptom-based prescreening that cannot capture asymptomatic cases, the prevalence should be systematically underestimated. However, B2 could also capture asymptomatic household members, and depending on the intrahousehold transmission rate, B2 could systematically overestimate the prevalence in certain scenarios. However, the average time between positive prescreening and LAMP result in B1 and B2 was 8.4 days; many of these participants had to be reminded to send the sample back. Hence, although an average duration of PCR positivity for 10 to 14 days seems reasonable, some people may have been positive when first receiving the study materials but cleared the infection before sampling [62]. In addition, the number of samples in B1 and B2 was considerably lower than that in A1 and A2 (1152 vs 4179 samples), which resulted in inaccurate prevalence estimates. Furthermore, the lower number of positive cases in B1 and B2 may have been because of the poor performance of the prescreening tool. The data sets used to train the random forest algorithm may not have been optimal to develop a sensitive discriminator. Including all household members in the prescreening could increase the chance of detecting symptomatic persons and might increase acceptance in parallel. However, this was omitted because of increased complexity and time constraints.

### Active Versus Passive Surveillance

In Germany, the RKI’s COVID-19 figures stem from passive surveillance, aiming to capture all positive test results (tested symptomatic persons and positively tested contact persons) to monitor the pandemic’s progress. However, data are transmitted through multiple tiers from physicians and laboratories to the RKI via local and state health departments, causing time delays that affect the official reporting of case numbers. The RKI therefore applies the nowcasting method for estimation by means of assumptions and imputation of missing values [3]. Asymptomatic cases are also underrepresented, making it challenging to assess the situation accurately. In contrast, an active surveillance system would not claim completeness of cases but test population representative random samples that represent the actual occurrence of infections. Furthermore, it would not rely on bottom-up reporting but sample infections directly with less delays and yielding more accurate prevalence estimates. Decentralized active surveillance systems could also detect regional differences timely. Furthermore, a Bayesian sliding-window estimator could enable the prediction of the number of hospitalizations above a certain prevalence threshold.

### Conclusions

The pandemic developed in a highly dynamic manner in most countries, with scarcely comparable trends. The succession of gradually increasing bouts of infection has been interrupted by periods of low infection activity. There is no reason to believe that future pandemics with similar hazard potential will evolve in a less chaotic manner; therefore, SARS-CoV-2 can be seen as a blueprint for future outbreaks. Therefore, rapidly deployable outbreak surveillance systems should be developed and put on standby.

Continuous active SARS-CoV-2 surveillance based on general population representative random samples of individuals or households and postal mailing of gargle sample collection kits for low-barrier home-based self-sampling, combined with a rapid, highly sensitive, and specific nucleic acid amplification test, is a feasible way to detect presymptomatic and asymptomatic cases without overburdening PCR capacities. However, additional measures are required to improve participation. Information material and consent processes should be tailored to real-life routine rather than scientific trials. In addition, the information material should be more concise and tested in pilot studies.

## Appendix

Multimedia Appendix 1 CONSORT (Consolidated Standards of Reporting Trials) 2010 checklist.

Multimedia Appendix 2 Supplementary materials, containing details on trial operationalization and certain calculations as well as supplementary tables and figures.

Multimedia Appendix 3 Study presentation, web-based conference on February 8, 2021.

## Abbreviations

CONSORT: Consolidated Standards of Reporting Trials
RKI: Robert Koch Institute
RT-LAMP: reverse transcription loop-mediated isothermal amplification
RT-PCR: real-time reverse transcription–polymerase chain reaction

## Data Availability

Anonymized data and a data dictionary will be made available in a public repository. Consent forms, invitation letters, and information material for the study are made available upon official request to the Network University Medicine for COVID-19 research in Germany. The study protocol has been published.
