# Correction: Comparison of Four Active SARS-CoV-2 Surveillance Strategies in Representative Population Sample Points: Two-Factor Factorial Randomized Controlled Trial

**DOI:** 10.2196/57203

**Published:** 2024-02-16

**Authors:** Andreas Deckert, Simon Anders, Ivonne Morales, Manuela De Allegri, Hoa Thi Nguyen, Aurélia Souares, Shannon McMahon, Matthias Meurer, Robin Burk, Dan Lou, Lucia Brugnara, Matthias Sand, Lisa Koeppel, Lena Maier-Hein, Tobias Ross, Tim J Adler, Stephan Brenner, Christopher Dyer, Konrad Herbst, Svetlana Ovchinnikova, Michael Marx, Paul Schnitzler, Michael Knop, Till Bärnighausen, Claudia M Denkinger

**Affiliations:** 1 Heidelberg Institute of Global Health Heidelberg Germany; 2 Center for Molecular Biology Heidelberg Heidelberg Germany; 3 Division of Infectious Disease and Tropical Medicine, Heidelberg University Hospital Heidelberg Germany; 4 evaplan GmbH at the University Hospital Heidelberg Germany; 5 GESIS Leibniz-Institute for the Social Sciences Mannheim Germany; 6 Division of Computer Assisted Medical Interventions, German Cancer Research Centre Heidelberg Germany; 7 Gesundheitsamt des Rhein-Neckar-Kreises Heidelberg Germany; 8 Center of Infectious Diseases, Virology, Heidelberg University Hospital Heidelberg Germany

In “Comparison of Four Active SARS-CoV-2 Surveillance Strategies in Representative Population Sample Points: Two-Factor Factorial Randomized Controlled Trial” (JMIR Public Health Surveill 2023;9:e44204) the authors noted one error.

In the originally published manuscript, the following sentence appeared in the “Study Design” section:

The 4 study arms represented all combinations of the two factors: (i) testing without condition (A) versus testing under the condition of upstream COVID-19 symptom prescreening (B) and (ii) testing of individuals (1) versus households (2) testing of individuals versus households.

This sentence has been corrected to:

The 4 study arms represented all combinations of two factors: i) testing unconditional (A) versus testing under the condition of upstream COVID-19 symptom prescreening (B), and ii) testing individuals (1) versus households (2).

The correction will appear in the online version of the paper on the JMIR Publications website on February 16, 2024, together with the publication of this correction notice. Because this was made after submission to PubMed, PubMed Central, and other full-text repositories, the corrected article has also been resubmitted to those repositories.

