# Supplementary material for: Comparison of Four Active SARS-CoV-2 Surveillance Strategies in Representative Population Sample Points: Two-Factor Factorial Randomized Controlled Trial
Source: JMIR Public Health Surveill. 2023 Aug 17;9:e44204. doi: 10.2196/44204 (PMC10437130; doi:10.2196/44204)

SUPPLEMENTARY MATERIAL

Details on trial operationalization and calculations

Trial implementation: The trial was completely designed and implemented within eight weeks only, including set up of logistics, random sampling, ethical approval, creation of a professional website and database, development of study information materials in German and translation of the website content into four additional languages (Turkish, English, Russian, Italian), et cetera. The actual conduct was limited to three weeks starting in mid of November 2020 in order to avoid an interaction with Christmas.

Randomized sampling: A stratified sample with two strata and two different sampling approaches was used for each of the three weekly batches. Stratum one only contained the city of Heidelberg, where potential participants were selected using a simple random sample from the municipality's population register. In stratum two, a two-stage sample design of the Rhine-Neckar district was applied. Here, at stage one, out of 55 municipalities 50 primary sampling units (PSU) were selected proportional to size, using the Cox algorithm. At stage two, the same number of secondary sampling units (SSU) for each sampled PSU was drawn, in order to obtain a self-weighted sample of individuals or households [42]. The net sample size was then translated into a corresponding gross sample under consideration of the expected response rates in each study arm and the sensitivity and specificity of the pre-screening tool applied to study arms B1 and B2 (see below). The gross sample therefore included a total of 21,739 individuals or households contacted in the Rhine-Neckar district and 6,386 in the "Heidelberg" stratum, separated into three weekly batches and subsequently into 15 consecutive recruitment weekdays [35]. Since with low probability more than one household member could have been selected in the municipalities' registers, we applied 10% oversampling to limit the number of initially contacted individuals to one person per household. Finally, the selected potential participants were randomly allocated to the four study arms.

The two-stage sampling was designed by the Leibnitz Institute for Social Sciences (GESIS) in Mannheim, Germany. Based on this, the HIGH requested the random samples from the municipalities through the local health authority.

Study materials and recruitment: In each of the study arms, the invitation letter explained the study purpose, the procedures, and the consent process and contained the declaration of consent. A comprehensive study brochure was enclosed, explaining the study and its conduct comprehensible for laypeople and containing instructions on how to produce a gargle sample and how to return the sample by mail. Additionally, the invitation letter contained simple links to the study website in five languages (German, Turkish, English, Russian, and Italian), a QR-code to access the gargling demonstration video on the website and the telephone number for the study hotline. The study information in the brochure and on the website as well as the consent forms were separately presented in adequate language for children from seven to 11, adolescents from 12 to 17, their parents, and adults.

The envelope with the invitation letter and the study information material also contained the pre-screening questionnaire for the initially contacted individuals (details see below). The completion of the pre-screening questionnaire was voluntary in the study arms A1 and A2, and a mandatory step before receiving a test kit in the study arms B1 and B2.

In study arm A1, the envelope additionally already contained a test kit, in A2 the test kits for the whole household. In the study arms B1 and B2 the pre-screening questionnaire was provided with the invitation letter without test kits. Here, the initially contacted individuals first had to complete the pre-screening questionnaire online or on paper. Only if the random forest analysis of the pre-screening data yielded a positive score, the initially contacted individual received a second envelope with test kit (B1), respectively, the test kits for the whole household (B2).

Children and adolescents were asked to assent their participation, with a mandatorily complemented consent of their legal representatives. Consent, respectively assent, to participate in the symptom screening could be given online or on paper, whereas consent, respectively assent, to get the gargle sample analyzed had to be provided on paper (see also supplementary material).

Study recruitment in the study arms B1 and B2 started on the 18 November 2020 and ended on the 8 December 2020, whereas recruitment in A1 and A2 started and ended half a week later. This shift warranted that the gargle liquid samples were taken in parallel within the four study arms since the participants in B1 and B2 had to fill out the pre-screening questionnaire first, which delayed the shipment of the test kits. One-time reminders were sent out until the 16 December 2020. The last questionnaires in the study arms B1 and B2 were accepted for analyses by the random forest algorithm on the 17 December, hence, the last test kits were sent out on the 18 December 2020. The last few gargle samples were received and analyzed on the 23 December 2020.

Pre-screening questionnaire: The pre-screening questionnaire consisted of 16 questions addressing the presence of COVID-19 symptoms, plus questions regarding the participants' socio-demographic background (i.e., education, employment, household size), the presence of some important chronic diseases such as diabetes and cardiovascular disease, and regular contact to children and patients. Only the symptoms were used in the pre-screening classifier. The questions on symptoms allowed the three answers "no", "yes", "often". For the questions about symptoms, the three possible answers were "no," "yes," and "often." The classifier counted "often" as "no" because this was then not considered an acute COVID-19 symptom.

The symptom screen was developed using state of the art machine learning algorithms to predict SARS-CoV-2 infection in the general population based on various symptoms. The underlying datasets were collected from various settings with and without SARS-CoV-2 infected patients, including samples from a general population screen and persons tested for SARS-CoV-2 due to COVID-19 symptoms or high-risk exposure. The original dataset was split into a training dataset (two third) and a test dataset (one third). Different machine learning algorithms were trained on the training dataset using 5-fold cross-validation and were evaluated on the test dataset. Their findings were compared and the final algorithm was chosen according to the best performance regarding the area under the curve (AUC). It constitutes an ensemble of two random forests algorithms to account for imputed missing data in the dataset.

The threshold of the algorithm was optimized towards 80% sensitivity of the receiver operating characteristic (ROC) curve leading to a specificity of 75%. Given the estimate of 0·5% prevalence of SARS-CoV-2 infection in the population when planning the trial, this yields a positive predictive value of 0·020 and a negative predictive value of 0·999.

Details on logistics: Address data received from the municipalities in encrypted tables were stored locally and only used for sending out the invitation and reminder letters. Only age, sex, municipality and the zip code were transferred into the subjects database. An automated event tracking system was used in order to allocate time stamps to each traceable event on individual level. For instance, each letter was scanned before it was sent out, and also incoming letters, questionnaires and samples were scanned right after their arrival. Each time a participant went online, the type of event such as filling out the online questionnaire, checking the results online or giving feedback were recorded. The date and time of hotline calls were also registered in the system, and the calls categorized and rated by the hotline staff. The subjects database contained a primary key which allowed the linkage with the other data tables.

Invitation letters without test kits in study arms B1 and B2 were packed and sent out daily (except for weekends), utilizing an external provider. Invitation letters containing test kits in study arms A1 and A2 were packed, the components scanned and linked, and sent out on a daily basis (except for weekends) by the study team. Second letters containing test kits in study arms B1 and B2 were prepared and sent out maximum 1 day after a questionnaire was filled out online or sent back via email, scanned and analyzed by the random forest algorithm. One-time reminder letters were generated semi-automatically with an online service of the German post based on automated daily lists if no event had been tracked within three to four days (considering weekends) after the invitation letter had been sent out.

Each participant received a unique 12-digit access code (including a security checksum) to request their test results online or at the hotline. The access code was linked to the barcode of the individual's vial for the gargle liquid sample by means of scanning both during packing of the consignments.

Once the gargle samples arrived, the consent forms were checked and digitalized. The bar codes of the vials were scanned and registered. The gargle samples were then processed by the laboratory on the same weekday. Incoming hardcopy questionnaires were also scanned immediately and digitalized. Unclear questionnaire scans were validated by assistants, using an online interface to the server.

The study hotline was functional for 25 working days and accessible on weekdays from seven to 11 am and from 2 to 6 pm. The hotline altogether received calls from 1782 contacted persons, with up to five calls per person, and an average of 80 calls per day, summing up to 2002 calls within the study period.

Self-sampling and RT-LAMP:

Real-time reverse transcriptase polymerase chain reaction (RT-PCR) is considered to be the gold standard for SARS-CoV-2 detection during acute infection and up to two to three weeks thereafter [20]. However, RT-PCR capacity is limited, thus, only applicable in population screening if pooling techniques are applied [21,22,43]. Additionally, the collection of nasopharyngeal (NP) or oropharyngeal swabs presents a logistical challenge, as medically trained personnel may be required. Specimens alternative to the gold-standard NP could achieve similar performance and enable self-sampling by probands and easy transportation [30]. Recent evidence suggests that gargle liquid, gargle solution, and cough mucus are suitable sources for the sensitive detection of SRAS-CoV-2 [26,28-33,44].

To enable scale-up of testing while maintaining sensitivity, a reverse transcription loop-mediated isothermal amplification-based method (RT-LAMP) is also applicable [45]. DNA primer sets are now available which allow the detection of viral RNA with similar sensitivity as RT-PCR [23-25]. The major advantage of RT-LAMP is its straightforward application which does not require a thermal cycler. Additionally, RT-LAMP is independent from the RT-PCR components' supply chain. Furthermore, the collection of gargle samples that also contain upper respiratory tract cells has been optimized in the meantime. The viral load in the gargle liquid hardly changes over several days when stored at room temperature which makes it even suitable for transport by post (20-25°C) [33].

Prevalence estimation: To assess the total number of SARS-CoV-2 cases among the population of the Rhine-Neckar region we used the Horvitz-Thomson estimator to correct for unequal sampling probabilities [40]. Probabilities for each person to get selected for participation in the study were based on the number of PSUs allocated to each municipality and the population in this municipality obtained from GESIS and were normalized for each study arm separately to match the number of performed tests. The Brewer approximation was used to calculate the estimate's variance [41]. Analyzing all the test results together yields an average prevalence during the study period. We have chosen not to employ a model-based non-response weighting or calibration for two reasons: 1) We cannot assume that the MAR (missing at random) assumption holds true. In sampling surveys, it is often the case that the MAR assumption is used by researchers, even when the underlying mechanism is actually NMAR (not missing at random). To address this issue, researchers typically incorporate numerous variables highly correlated with the response mechanism. 2) Unfortunately, we were unable to establish the precise relationship between (the probability of) non-response, our variable(s) of interest, and auxiliary variables. Consequently, the risk of misspecification was higher. Therefore, relying solely on the unscaled design weights to determine the absolute (and relative) quantity of positive tests can be considered a more conservative approach. For arms B1 and B2, all the participants who submitted a questionnaire, but were classified negative for SARS-CoV-2 infection due to the absence of relevant COVID-19 symptoms were also included in these estimates. For arm B2 in addition the reported number of household members was used. Since this approach does not include asymptomatic cases and assumes 100% sensitivity of the pre-screening classifier, the obtained prevalence value is expected to be below the true one. We also calculated the fraction of positive cases among all performed tests in arms B1 and B2. These values are expected to be above the true prevalence value and can be used to estimate the specificity of the classifier. To combine the results of the arms A1 and A2 or B1 and B2 we subset our dataset to include only the initially contacted participants.

Prevalence estimate based on the reported cases of the RKI: To compare the results of our study with the reported number of cases, we estimated the reported prevalence based on data from the Robert Koch-Institute (RKI), which provides daily updated data for download online [46]. Derived from these data we calculated the incidence for any given period. However, to get prevalence estimates one needs to additionally know the disease duration. Hereafter, we call "duration" the period when viral RNA can be detected in an infected person's sample and therefore the person can be considered infected based on the RT-PCR test result. For the sake of simplicity, we will call infected people infected during this period, even though real symptoms may persist for a longer time. For their reports and web dashboard, RKI uses the following durations: 14 days for mild/asymptomatic cases and 28 days for hospitalized and/or severe cases [46]. Though even longer durations are possible, overall such assumption is probably an overestimate of the disease duration [47]. Moreover, it is not recorded, whether a certain case was mild or severe. However, it is known that the COVID-19 duration varies a lot depending on the severity of the disease. Yet, to calculate the prevalence based on the incidence data we assumed a fixed average duration for all the cases, which balances out long-lasting infections with short ones.

Presuming we could exactly measure the point prevalence every day during some period. Every infected person then adds one case-day to this estimate every day he or she is infected. For the prevalence estimate it does not matter whether this additional one case-day stems from the same person as the day before. Thus, we can redistribute those "plus ones" so that every person is counted the number of times equal to the average duration in days, thus, keeping the total number of "plus ones" over the period the same. Such an approach provides a somewhat smoothed prevalence estimate over time.

Assuming that f_j is the fraction of infected people, who remain sick for j days and $\sum_j f_j = 1$. i_l is incidence on day l. Then the total number of cases on day l is:

$$t_l = \sum_{k=0}^{l-1} \left(i_{l-k} \sum_{j>k} f_j \right) = \sum_j \left(f_j \sum_{k=0}^{j-1} i_{l-k} \right) = \sum_j j \cdot f_j \cdot \overline{\imath_{(l-j+1):l}}$$

where $\overline{\imath_{a:b}}$ is an average incidence between days a and b.

The average duration is

$$d = \sum_j j \cdot f_j$$

And in simplification, the total number of cases on day l is calculated as

$$\widehat{t_l} = \sum_{k=0}^{d-1} i_{l-k} = d \cdot \overline{\iota_{(l-d+1):l}} = \sum_j j \cdot f_j \cdot \overline{\iota_{(l-d+1):l}}$$

If incidence does not change, the two estimates t_l and $\widehat{t_l}$ are exactly the same. If incidence changes (as in our case, for, example it grows), $\widehat{t_l}$ is still a reasonable estimation for t_l, since we overestimate the average incidence with $\overline{\iota_{(l-d+1):l}}$ for longer durations and compensate for it by underestimating it for shorter durations.

Since there is a huge variation in the reported average duration of a SARS-CoV-2 infection [47], we made comparisons for different durations within a reasonable range.

Simulating study results based on known prevalence: We additionally provide an alternative approach to compare the estimated prevalence in our study with the reported number of cases. For every day of the study, we calculated the prevalence estimate based on the RKI data as described above. Then we used the number of analyzed samples on that day to estimate how many positive cases we should have detected based on the calculated prevalence value. Since many asymptomatic cases are missing in the RKI data and also the symptomatic cases are incomplete, the prevalence derived from these data should be lower than the estimates in the study arms A1 and A2. In other words, the study prevalence estimates above the estimated prevalence based on RKI reported number of cases provide an insight into the amount of cases missed by the passive surveillance system.

The total number of tests for each assumed average duration was estimated as a sum of several binomial distributions (one for each day of the study) with the prevalence based on the RKI data as their probabilities. We simulated the total number by drawing it from this simple and well-defined distribution 500 times for each duration. In N=312 cases (5·8%) the sampling date was not provided by the participants and we used the middle date between the dates when the test kit was sent out and the date when the sample was received by the lab, when calculating the number of samples taken on each day.

Sensitivity analysis: The trial was implemented in parallel to the existing passive surveillance system, hence, persons could have been captured by both systems. Actually, some hotline callers refused to participate since they already had been tested positive elsewhere, however, these individuals would have been captured by the trial as well. Consequently, we conducted sensitivity analysis and re-calculated the prevalence estimates. For this, we matched hotline cases with the COVID-19 database of the local health authority (secondary cases excluded). We then calculated the difference between the date the test kit has been dispatched or alternatively, the date when the person called the hotline after receiving the invitation letter in B1/B2 and the PCR test date as recorded in the COVID-19 database of the local health authority. We considered cases where the PCR test was conducted 14 days prior to the test kit dispatch or the hotline call.

We additionally checked how many of all non-responders were known positive cases by the local health authority within 14 days prior to the dispatch of the invitation letter. To fuzzy match the records of the study with the COVID-19 database of the local health authority, a Python package called Fuzzy Wuzzy that measures the Levenshtein distance - the distance between A and B in terms of how many changes are made to string A to transform it into string B (changes include removing, adding or substituting characters) was applied [48]. Essentially, a string containing the age, name, address, gender and postcode for each record in both datasets was prepared, and then the comparison scored. Afterwards, additional semi-automated checks were run to manually remove fairly obvious mismatches. As in accordance with the data protection laws, the researchers only received summary statistics from the local health authority.

Supplementary tables and graphs

Figure S1: Randomized, two-factor factorial, four-group parallel study design

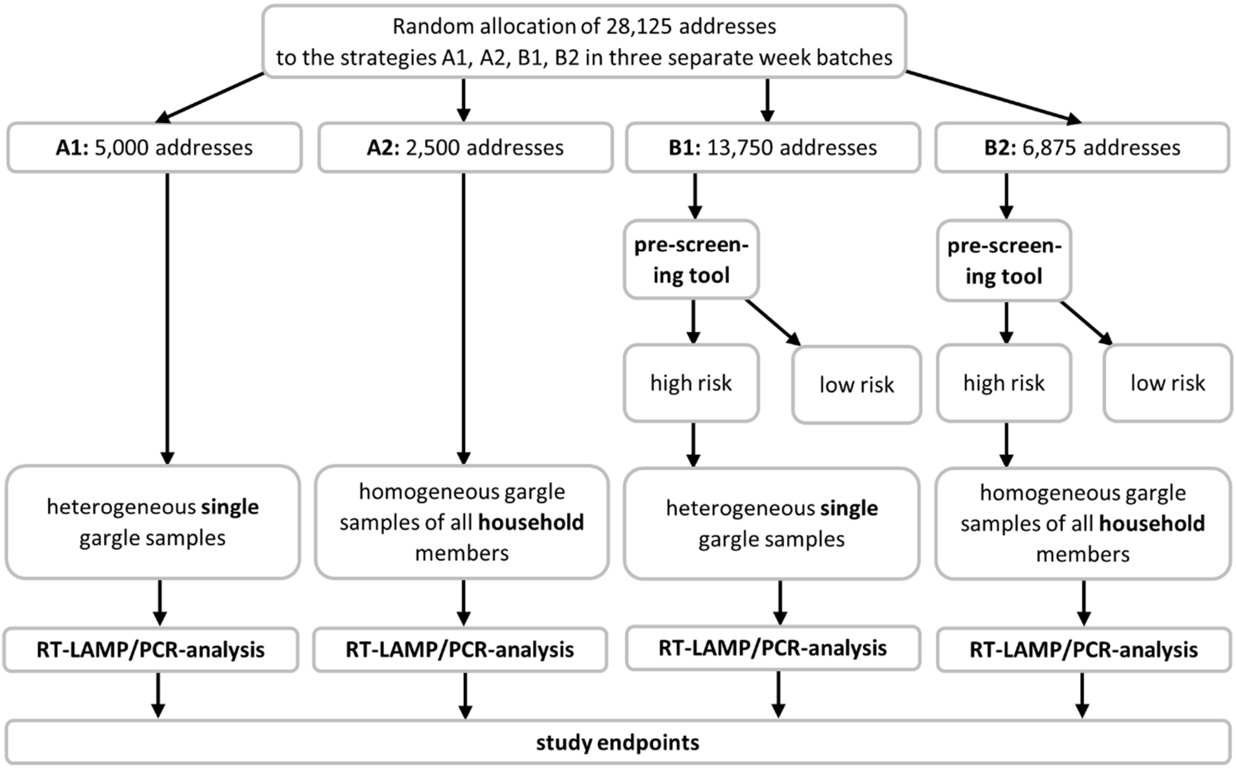

		A1<br>N=4962 (%)	A2<br>N=2481 (%)	B1<br>N=13644 (%)	B2<br>N=6822 (%)	Total<br>N=27909 (%)
**Sex**	Female	2469 (49·8)	1248 (50·3)	7021 (51·5)	3478 (51·0)	14216 (50·9)
	Male	2493 (50·2)	1233 (49·7)	6623 (48·5)	3344 (49·0)	13693 (49·1)
**Age [years]**	Mean	45·2	46·0	45·2	45·4	45·3
	Range	7-101	7-98	7-100	7-100	7-101

Table S1: Demographics of initial random sample (individuals randomly drawn from the 51 municipalities' population registers and randomly allocated to the study arms before dispatching invitation letters)

		A1<br>N=2088 (%)	A2<br>N=2288 (%)	B1<br>N=4926 (%)	B2<br>N=2677 (%)	Total<br>N=11919 (%)	p-value
**Sex**	Female	1089 (52·2)	1081 (48·5)	2615 (53·1)	1425 (53·2)	6210 (52·1)	< 0·001 [1]
	Male	999 (47·8)	1069 (48·0)	2311 (46·9)	1225 (45·8)	5604 (47·0)	
	N-Miss	0 (0·0)	78 (3·5)	0 (0·0)	27 (1·0)	105 (0·1)	
**Age [years]**	Mean	46·7	44·2	47·2	47·1	46·6	< 0·001 [2]
	Range	7 – 99	7 – 97	7 – 98	7 – 98	7 – 99	
	N-Miss	0 (0·0)	94 (4·2)	0 (0·0)	27 (1·0)	121 (1·0)	

[1] Pearson's Chi-squared test, [2] Linear Model ANOVA

Table S2: Demographics of any responder (A1, B1: initially contacted individuals s who provided a valid sample and/or a valid questionnaire; A2, B2: initially contacted individuals who provided a valid sample and/or a valid questionnaire plus household members who provided a valid sample; age and sex were available for initially contacted individuals from the registration offices and for household members from the consent form)

Symptoms		A1, N=1942 (%)	A2, N=764 (%)	B1, N=4926 (%)	B2, N=2340 (%)	Total (N=9972)	p value[1]
**Fever**	Yes	113 (5·8)	31 (4·1)	200 (4·1)	111 (4·7)	455 (4·6)	0·012
	No	1810 (93·2)	724 (94·8)	4722 (95·9)	2226 (95·1)	9482 (95·1)	
	Missing	19 (1·0)	9 (1·2)	4 (0·1)	3 (0·1)	35 (0·4)	
**Cough**	Yes	242 (12·5)	75 (9·8)	580 (11·8)	286 (12·2)	1183 (11·9)	0·269
	No	1673 (86·1)	676 (88·5)	4328 (87·9)	2043 (87·3)	8720 (87·4)	
	Missing	27 (1·4)	13 (1·7)	18 (0·4)	11 (0·5)	69 (0·7)	
**Cough with phlegm/mucus**	Yes	213 (11·0)	71 (9·3)	467 (9·5)	226 (9·7)	977 (9·8)	0·224
	No	1704 (87·7)	686 (89·8)	4448 (90·3)	2109 (90·1)	8947 (89·7)	
	Missing	25 (1·3)	7 (0·9)	11 (0·2)	5 (0·2)	48 (0·5)	
**Sore throat**	Yes	207 (10·7)	71 (9·3)	513 (10·4)	235 (10·0)	1026 (10·3)	0·713
	No	1712 (88·2)	684 (89·5)	4406 (89·4)	2101 (89·8)	8903 (89·3)	
	Missing	23 (1·2)	9 (1·2)	7 (0·1)	4 (0·2)	43 (0·4)	
**Difficulty breathing**	Yes	151 (7·8)	47 (6·2)	291 (5·9)	152 (6·5)	641 (6·4)	0·030
	No	1763 (90·8)	709 (92·8)	4624 (93·9)	2180 (93·2)	9276 (93·0)	
	Missing	28 (1·4)	8 (1·0)	11 (0·2)	8 (0·3)	55 (0·6)	
**Muscle aches**	Yes	231 (11·9)	81 (10·6)	626 (12·7)	287 (12·3)	1225 (12·3)	0·452
	No	1684 (86·7)	674 (88·2)	4293 (87·1)	2047 (87·5)	8698 (87·2)	
	Missing	27 (1·4)	9 (1·2)	7 (0·1)	6 (0·3)	49 (0·5)	
**Exhaustion**	Yes	447 (23·0)	144 (18·8)	1225 (24·9)	548 (23·4)	2364 (23·7)	0·004
	No	1473 (75·8)	614 (80·4)	3690 (74·9)	1786 (76·3)	7563 (75·8)	
	Missing	22 (1·1)	6 (0·8)	11 (0·2)	6 (0·3)	45 (0·5)	
**Headache**	Yes	311 (16·0)	89 (11·6)	813 (16·5)	399 (17·1)	1612 (16·2)	0·005
	No	1600 (82·4)	668 (87·4)	4105 (83·3)	1937 (82·8)	8310 (83·3)	
	Missing	31 (1·6)	7 (0·9)	8 (0·2)	4 (0·2)	50 (0·5)	
**Runny nose**	Yes	379 (19·5)	151 (19·8)	953 (19·3)	494 (21·1)	1977 (19·8)	0·385
	No	1537 (79·1)	607 (79·5)	3965 (80·5)	1845 (78·8)	7954 (79·8)	
	Missing	26 (1·3)	6 (0·8)	8 (0·2)	1 (0·0)	41 (0·4)	
**Chest pain**	Yes	135 (7·0)	48 (6·3)	303 (6·2)	169 (7·2)	655 (6·6)	0·280
	No	1780 (91·7)	710 (92·9)	4619 (93·8)	2166 (92·6)	9275 (93·0)	
	Missing	27 (1·4)	6 (0·8)	4 (0·1)	5 (0·2)	42 (0·4)	
**Diarrhea**	Yes	160 (8·2)	45 (5·9)	391 (7·9)	180 (7·7)	776 (7·8)	0·204
	No	1756 (90·4)	712 (93·2)	4528 (91·9)	2153 (92·0)	9149 (91·7)	
	Missing	26 (1·3)	7 (0·9)	7 (0·1)	7 (0·3)	47 (0·5)	
**Nausea**	Yes	114 (5·9)	35 (4·6)	237 (4·8)	116 (5·0)	502 (5·0)	0·243
	No	1799 (92·6)	723 (94·6)	4684 (95·1)	2218 (94·8)	9424 (94·5)	
	Missing	29 (1·5)	6 (0·8)	5 (0·1)	6 (0·3)	46 (0·5)	
**Ageusia/ Anosmia**	Yes	123 (6·3)	36 (4·7)	238 (4·8)	112 (4·8)	509 (5·1)	0·043
	No	1793 (92·3)	722 (94·5)	4682 (95·0)	2221 (94·9)	9418 (94·4)	
	Missing	26 (1·3)	6 (0·8)	6 (0·1)	7 (0·3)	45 (0·5)	
**Chills**	Yes	113 (5·8)	31 (4·1)	208 (4·2)	112 (4·8)	464 (4·7)	0·026
	No	1803 (92·8)	726 (95·0)	4713 (95·7)	2223 (95·0)	9465 (94·9)	
	Missing	26 (1·3)	7 (0·9)	5 (0·1)	5 (0·2)	43 (0·4)	
**Short breath**	Yes	191 (9·8)	73 (9·6)	473 (9·6)	247 (10·6)	984 (9·9)	0·623
	No	1723 (88·7)	684 (89·5)	4449 (90·3)	2086 (89·1)	8942 (89·7)	
	Missing	28 (1·4)	7 (0·9)	4 (0·1)	7 (0·3)	46 (0·5)	
**Confusion**	Yes	111 (5·7)	27 (3·5)	217 (4·4)	103 (4·4)	458 (4·6)	0·034
	No	1802 (92·8)	730 (95·5)	4701 (95·4)	2229 (95·3)	9462 (94·9)	
	Missing	29 (1·5)	7 (0·9)	8 (0·2)	8 (0·3)	52 (0·5)	

[1] Pearson's Chi-squared test

Table S3: Covid-19 symptoms among those who filled out the questionnaire (initially contacted individuals only)

Figure S2: Frequencies of present Covid-19 related symptoms among those who filled out the pre-screening questionnaire and scored positive (initially contacted individuals only; A1 and A2 voluntary, B1 and B2 mandatory)

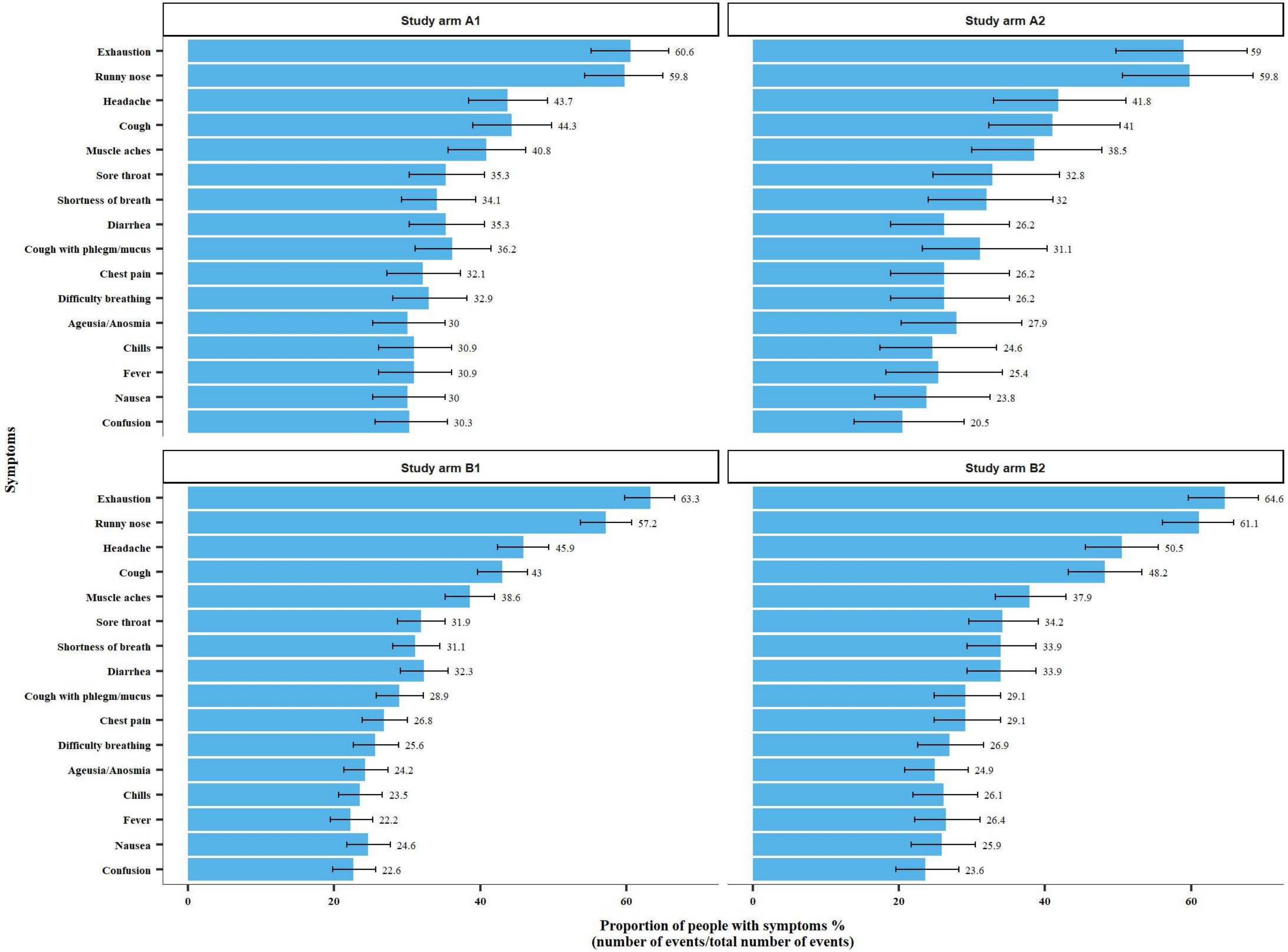

Figure S3: Distribution of number of COVID-19 symptoms among positively and negatively screened initially contacted individuals, as assessed by the random forest algorithm

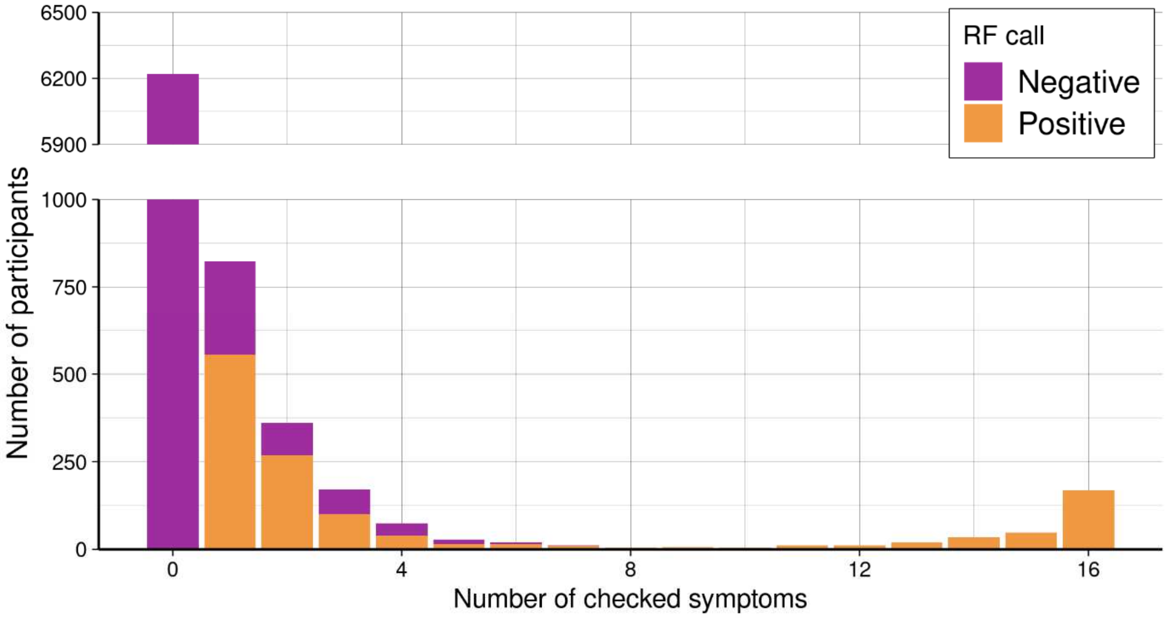

		A1 N=1942 (%)	A2 N=764 (%)	B1 N=4926 (%)	B2 N=2340 (%)	Total N=9972 (%)	p-value[1]
**Flu vaccination**	Yes	656 (33·8)	275 (36·0)	1685 (34·2)	845 (36·1)	3461 (34·7)	0·277
	No	1183 (60·9)	455 (59·6)	3131 (63·6)	1441 (61·6)	6210 (62·3)	
	Missing	103 (5·3)	34 (4·5)	110 (2·2)	54 (2·3)	301 (3·0)	
**Works with children**	Yes	251 (12·9)	107 (14·0)	677 (13·7)	318 (13·6)	1353 (13·6)	0·916
	No	1516 (78·1)	592 (77·5)	3963 (80·5)	1881 (80·4)	7952 (79·7)	
	Missing	175 (9·0)	65 (8·5)	286 (5·8)	141 (6·0)	667 (6·7)	
**Performs medical-related work**	Yes	145 (7·5)	52 (6·8)	340 (6·9)	164 (7·0)	701 (7·0)	0·724
	No	1646 (84·8)	656 (85·9)	4337 (88·0)	2053 (87·7)	8692 (87·2)	
	Missing	151 (7·8)	56 (7·3)	249 (5·1)	123 (5·3)	579 (5·8)	
**Asthma**	Yes	142 (7·3)	53 (6·9)	351 (7·1)	159 (6·8)	705 (7·1)	0·931
	No	1797 (92·5)	705 (92·3)	4538 (92·1)	2166 (92·6)	9206 (92·3)	
	Missing	3 (0·2)	6 (0·8)	37 (0·8)	15 (0·6)	61 (0·6)	
**Other lung problems**	Yes	111 (5·7)	44 (5·8)	259 (5·3)	134 (5·7)	548 (5·5)	0·806
	No	1829 (94·2)	715 (93·6)	4628 (94·0)	2191 (93·6)	9363 (93·9)	
	Missing	2 (0·1)	5 (0·7)	39 (0·8)	15 (0·6)	61 (0·6)	
**Diabetes**	Yes	104 (5·4)	35 (4·6)	180 (3·7)	78 (3·3)	397 (4·0)	0·001
	No	1751 (90·2)	706 (92·4)	4695 (95·3)	2236 (95·6)	9388 (94·1)	
	Missing	87 (4·5)	23 (3·0)	51 (1·0)	26 (1·1)	187 (1·9)	
**High blood pressure**	Yes	387 (19·9)	133 (17·4)	604 (12·3)	281 (12·0)	1405 (14·1)	< 0·001
	No	1471 (75·7)	610 (79·8)	4272 (86·7)	2032 (86·8)	8385 (84·1)	
	Missing	84 (4·3)	21 (2·7)	50 (1·0)	27 (1·2)	182 (1·8)	
**Other cardiovascular diseases**	Yes	98 (5·0)	34 (4·5)	153 (3·1)	84 (3·6)	369 (3·7)	< 0·001
	No	1750 (90·1)	710 (92·9)	4714 (95·7)	2228 (95·2)	9402 (94·3)	
	Missing	94 (4·8)	20 (2·6)	59 (1·2)	28 (1·2)	201 (2·0)	
**Chronic kidney disease**	Yes	26 (1·3)	6 (0·8)	38 (0·8)	23 (1·0)	93 (0·9)	0·125
	No	1826 (94·0)	736 (96·3)	4831 (98·1)	2294 (98·0)	9687 (97·1)	
	Missing	90 (4·6)	22 (2·9)	57 (1·2)	23 (1·0)	192 (1·9)	
**Any cancer in the last 2 years**	Yes	40 (2·1)	12 (1·6)	72 (1·5)	41 (1·8)	165 (1·7)	0·273
	No	1812 (93·3)	792 (95·4)	4799 (97·4)	2276 (97·3)	9616 (96·4)	
	Missing	90 (4·6)	23 (3·0)	55 (1·1)	23 (1·0)	191 (1·9)	

[1] Pearson's Chi-squared test

Table S4: Work and co-morbidity data from the pre-screening questionnaire (initially contacted individuals only)

Study arm	Without hotline cases, mean (95% CI)	With hotline cases, mean (95% CI)
A1 [a]	0·32% (0·06% – 0·58%)	0·37% (0·10% – 0·65%)
A2 [a]	0·35% (0·09% – 0·61%)	0·35% (0·09% – 0·61%)
A [a,c]	0·36% (0·14% – 0·59%)	0·40% (0·16% – 0·64%)
B1 [a]	0·07% (0·00% – 0·15%)	0·14% (0·03% – 0·26%)
B2 [a]	0·02% (0·00% – 0·06%)	0·07% (0·00% – 0·15%)
B [a]	0·05% (0·00% – 0·10%)	0·11% (0·03% – 0·19%)
B1 [b]	0·59% (0·00% – 1·26%)	1·18% (0·24% – 2·13%)
B2 [b]	0·19% (0·00% – 0·57%)	0·72% (0·02% – 1·41%)
B [b,c]	0·40% (0·00% – 0·86%)	0·93% (0·24% – 1·62%)

[a] prevalence estimate
[b] fraction of positive cases among all samples analyzed with PCR
[c] only initial cases are counted

Table S5: Estimated prevalence, calculated for each arm separately, and for arms A1 and A2 (arm A) and B1 and B2 (arm B) pooled together. For arms B1 and B2 additional the fraction of positive cases is included. For combined arms A and B only initially contacted participants are counted to avoid household bias.

Figure S4: Official numbers of daily new cases for the Rhine-Neckar region in 2020 and during the Cov Surv study (indicated by the vertical lines), as reported by the Robert Koch Institute (RKI)

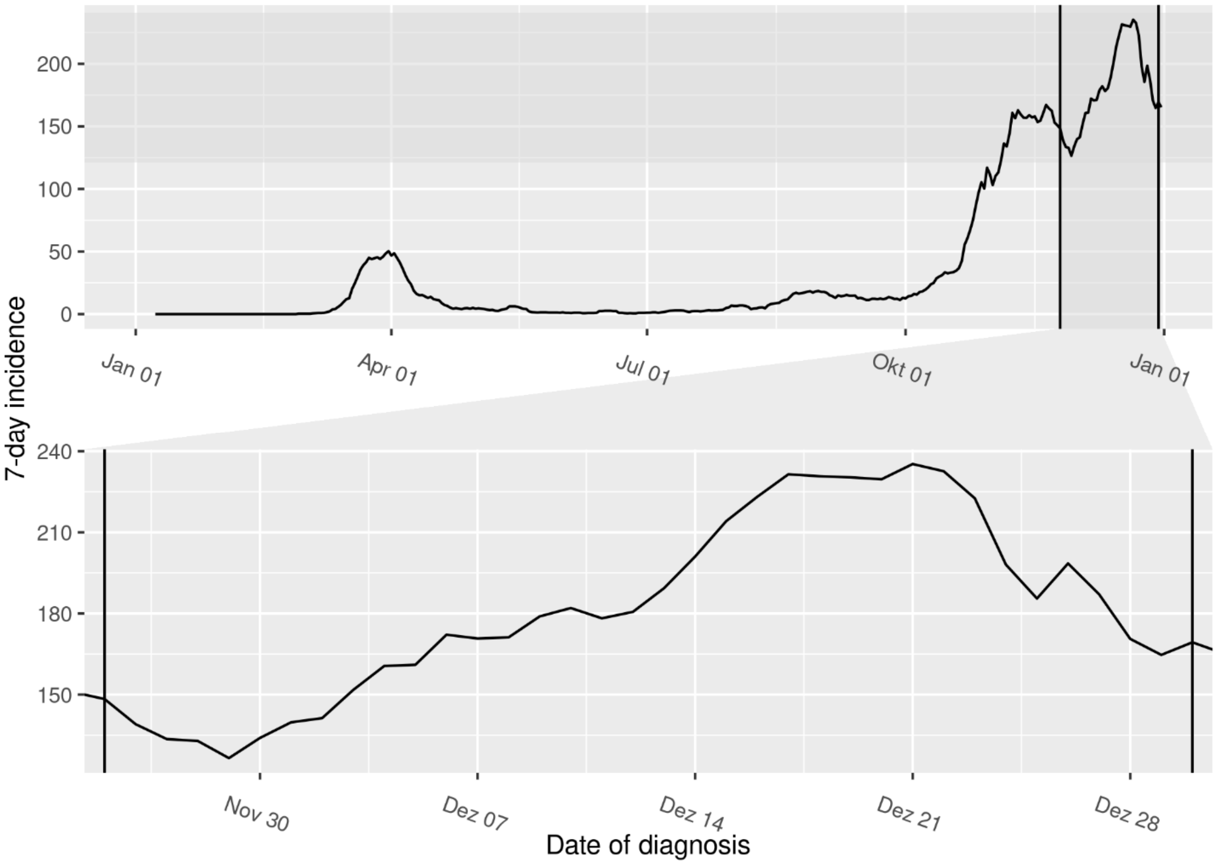

Figure S5: Simulated number of positive cases that should have been detected at minimum in arms A1 and A2, considering RKI data constitutes the lower bound of the prevalence estimate. (The purple line displays the simulated number depending on the assumed average duration of an infected person having a detectable amount of SARS-CoV2 RNA in the sample. Shaded area corresponds to the 95% confidence interval.)

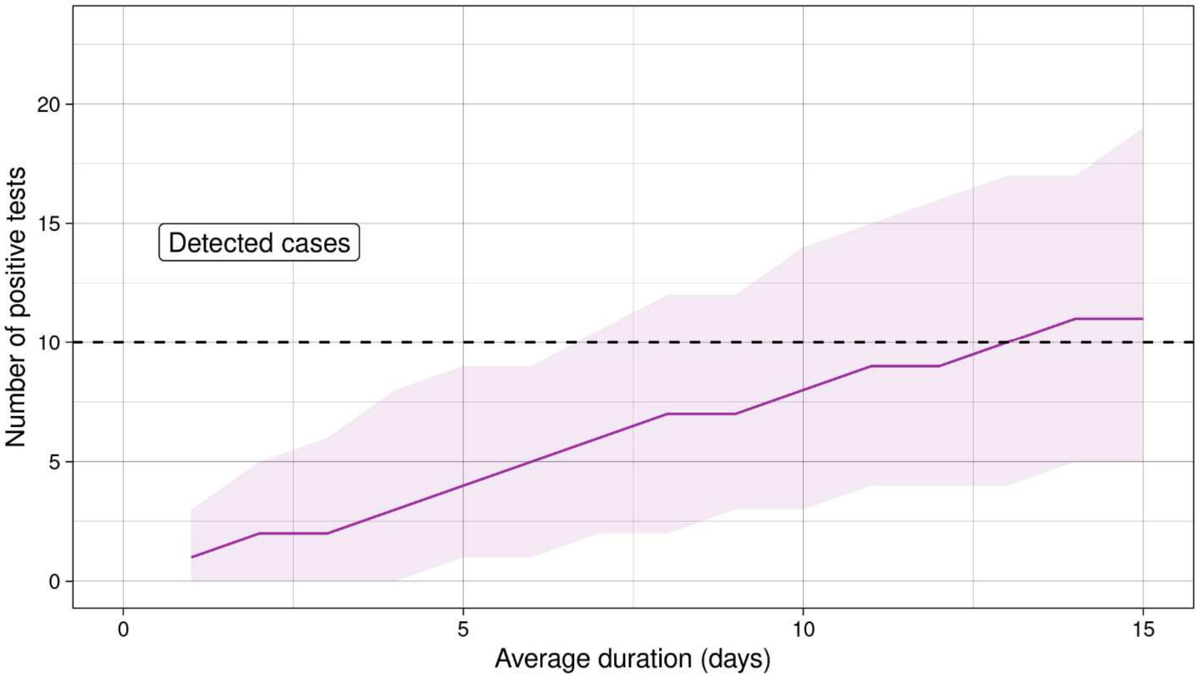

Explanation: To get exact prevalence estimates based on the RKI data, we drew the number of positive tests 500 times for each disease duration, day of the study, and Heidelberg, respectively, Rhine-Neckar district (data provided separately) from the corresponding binomial distribution. We then used the median value as the number of expected positive tests. The dashed line corresponds to the median value of all the simulated values, with the red area containing 95%. The horizontal black line is the overall number of positive samples in the study. The blue horizontal lines are 95% confidence interval for a binomial distribution with the estimated prevalence as the probability.

Figure S6: Estimates for prevalence for each arm with the hotline cases included. Light blue in addition shows the fraction of positive cases among all the performed tests. For combined arms A and B only the initially contacted participants are included.

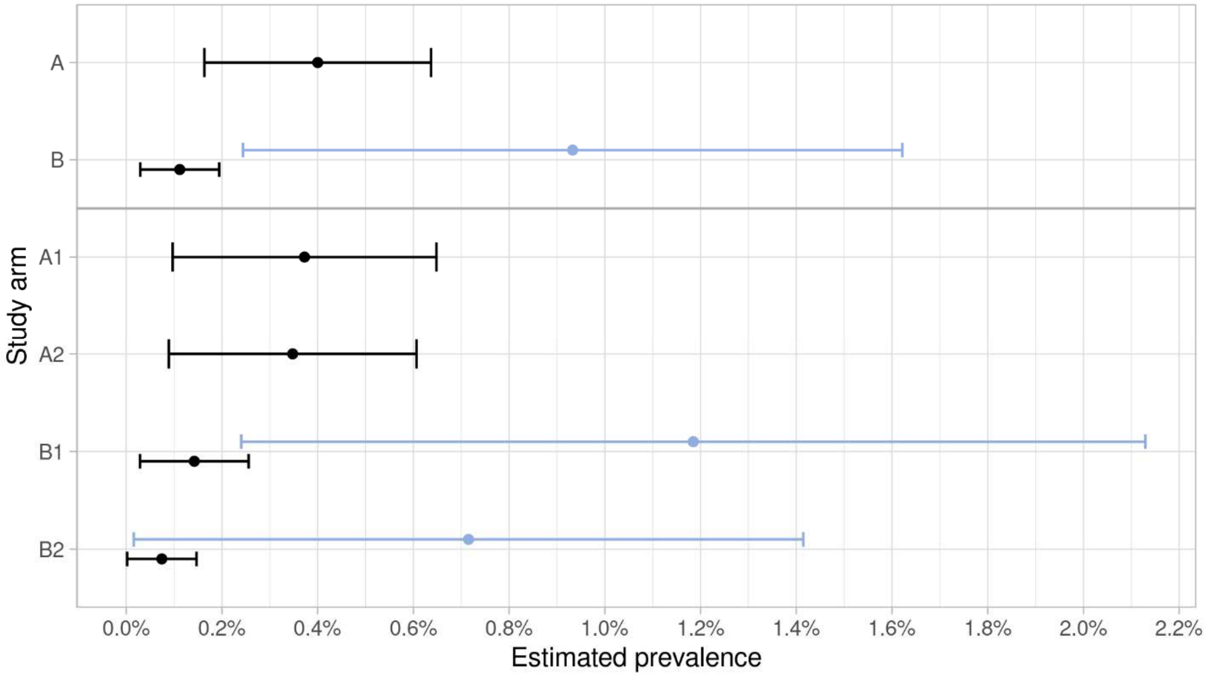

Supplement: Multimedia Appendix 2 [file publichealth_v9i1e44204_app2.pdf]
