# Supplementary material for: Comparison of Four Active SARS-CoV-2 Surveillance Strategies in Representative Population Sample Points: Two-Factor Factorial Randomized Controlled Trial
Source: JMIR Public Health Surveill. 2023 Aug 17;9:e44204. doi: 10.2196/44204 (PMC10437130; doi:10.2196/44204)

This is a **Multimedia Appendix** to a full manuscript published in the J Med Internet Res.
For full copyright and citation information see http://dx.doi.org/10.2196/jmir.44204

UNIVERSITÄTS
KLINIKUM
HEIDELBERG

B-FAST
bundesweites forschungsnetz
angewandte surveillance und testung

VIRUSFINDER
FÜR DICH. FÜR UNS. FÜR ZUKUNFT.

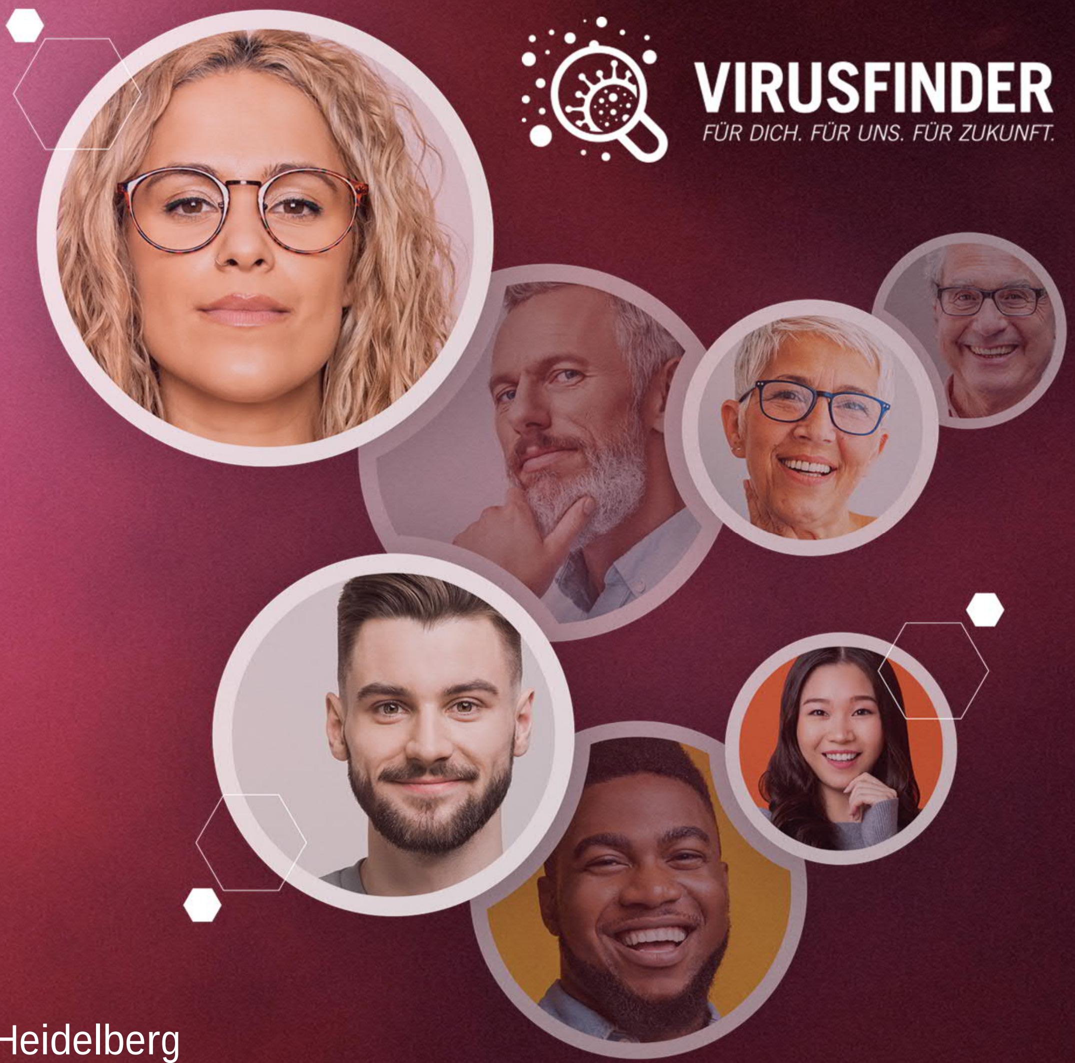

Cluster-randomised 2-factorial controlled study to test the (cost-) effectiveness of four SARS-CoV-2 surveillance-strategies for the general population

Dr. Andreas Deckert
Heidelberg Institute of Global Health
08. February 2021, online partnership meeting Kumamoto - Heidelberg

Region Rhine-Neckar and Heidelberg

VIRUSFINDER
FÜR DICH. FÜR UNS. FÜR ZUKUNFT.

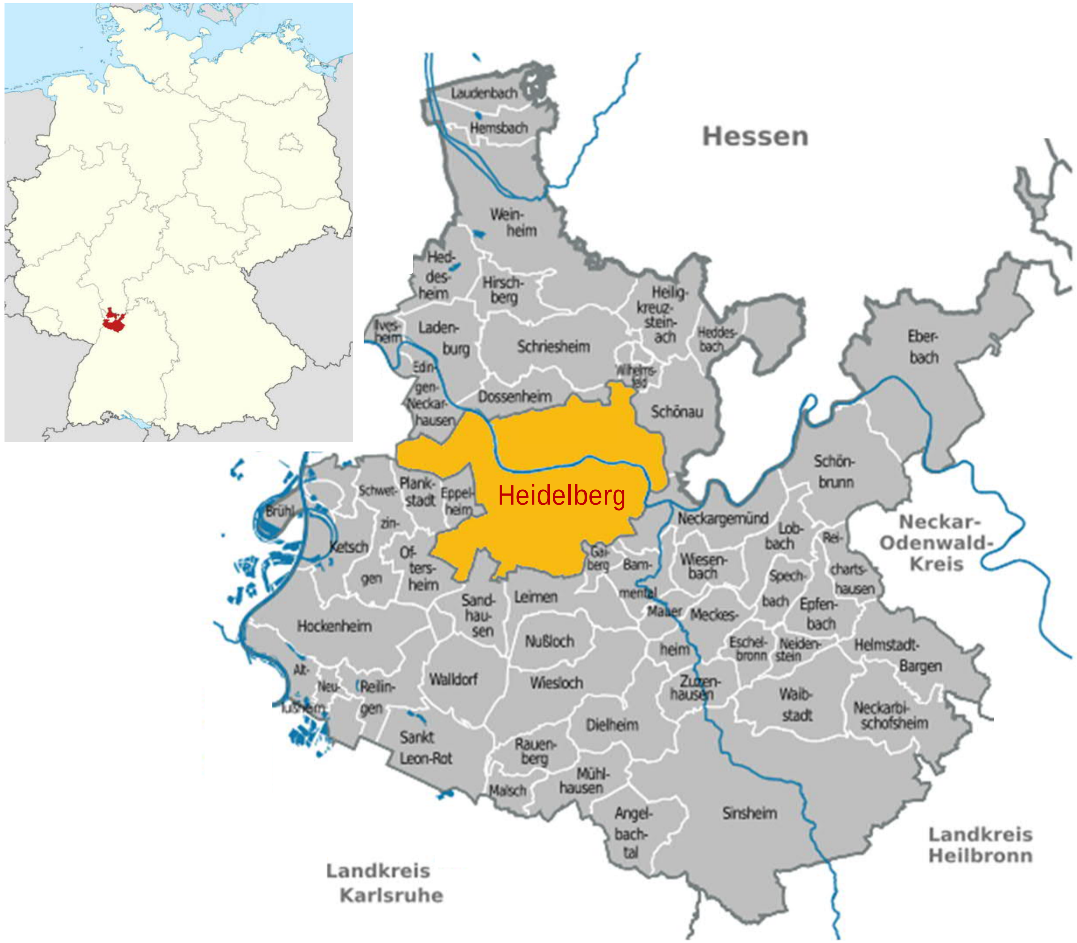

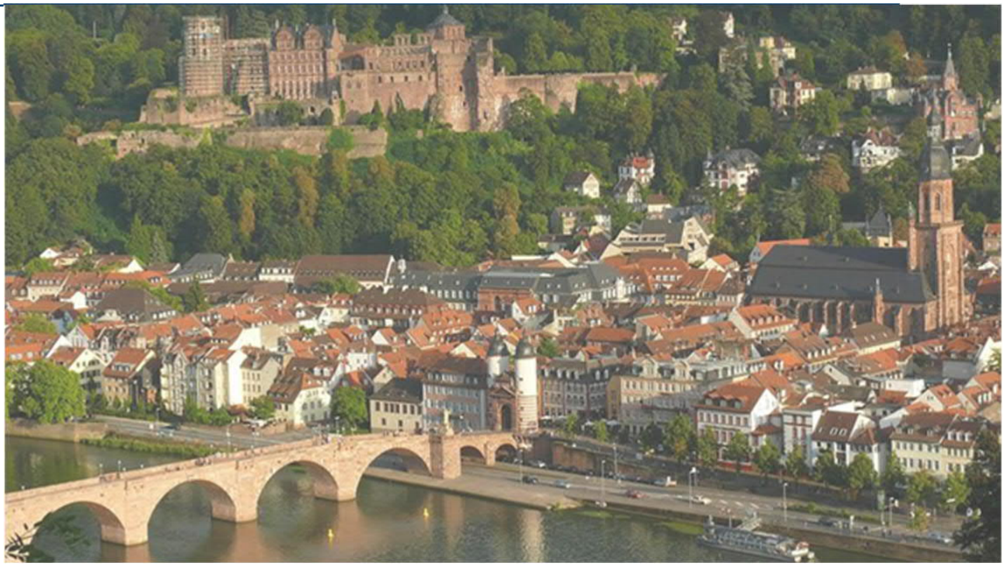

- Rhine-Neckar region: around 550,000 residents, fifth largest district in Germany
- Heidelberg: around 160,000 residents

→ Catchment area *Virusfinder* around 700,000 residents

ZMBH
UKHD

Surveillance Rhein-Neckar: CRCT, 2-factorial design

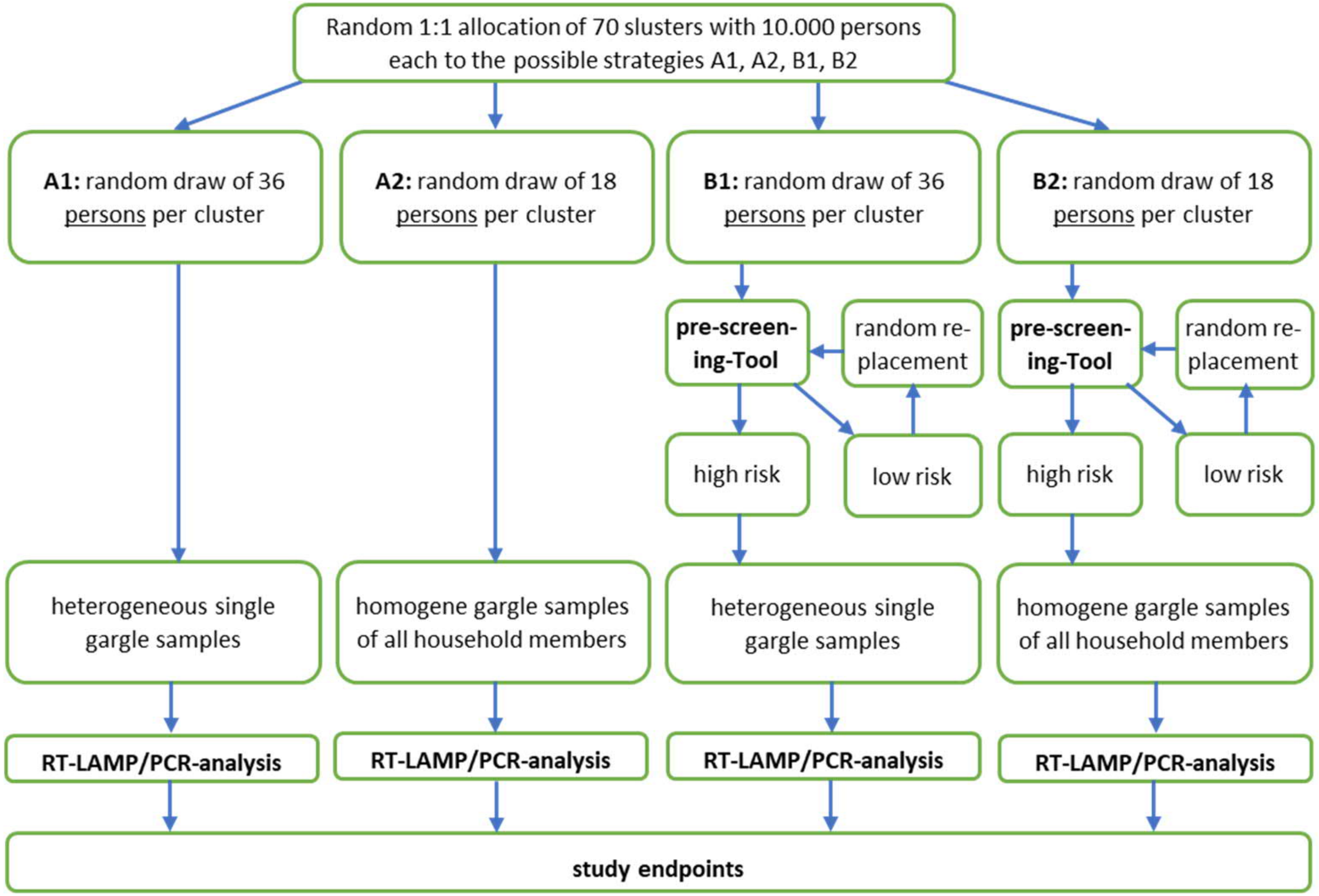

Assumptions:

- Response 50%
- After pre-screening 80%
- Screening sens. 90%, spec. 70%
- 4-week prevalence 0,5%
- With 2500 tests 80% power for a prevalence of 0,5%±0,33

→ Samples:

- A1 (single): 5000
- A2 (household): 2500
- B1 (single): 13750
- B2 (houshold): 6875

Response

	Direct gargle sampling		Symptom pre-screening	
	A1, single	A2, household	B1, single	B2 , household
Final study sample	4962 (99%)	2481 (99%)	13644 (99%)	6822 (99%)
Response rate	2085 (42%)	901 (36%)	5065 (37%)	2413 (35%)
Added household members	n.a.	1376	n.a.	362 [a]
Completed questionnaires („pre-screening“)	*1868 (90%)*	*792 (88%)*	5065 (100%)	2413 (100%)
Valid gargle samples	2033 (96%)	2207 (97%)	557 (72%) [b]	245 (65%) [c]
SARS-CoV-2 diagnosis (after PCR confirmation test) [d]	7	4	3	0
SARS-CoV-2 diagnosis household members	n.a.	3	n.a.	1

[a] Questionnaire completed only by initial contacts, if pre-screening positive, then gargle samples were requested from all household members

[b] Related to positive pre-screening; out of 5065 pre-screening questionnaires 777 (15%) were suspicious

[c] Related to positive pre-screening; out of 2413 pre-screening questionnaires 377 (16%) were supsicious

[d] Additionally, 3 positive reports on the hotline without participation in B2, 1 positive report without disclosure of study arm

→ 3 week cumulative SARS-CoV-2 prevalence in A1, A2 (unweighted): around 0.3% each

Implementation: B(e)-FAST!

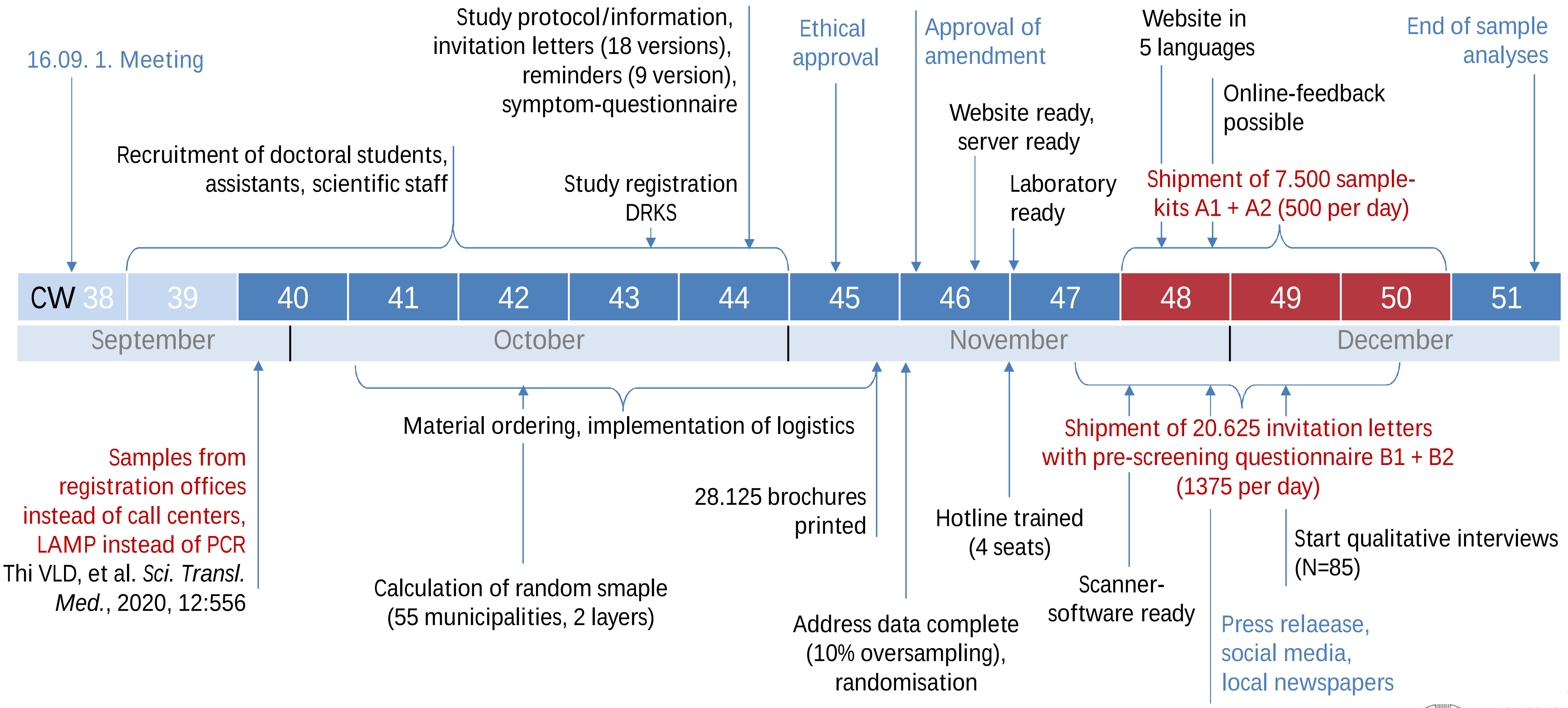

Sampling logistics

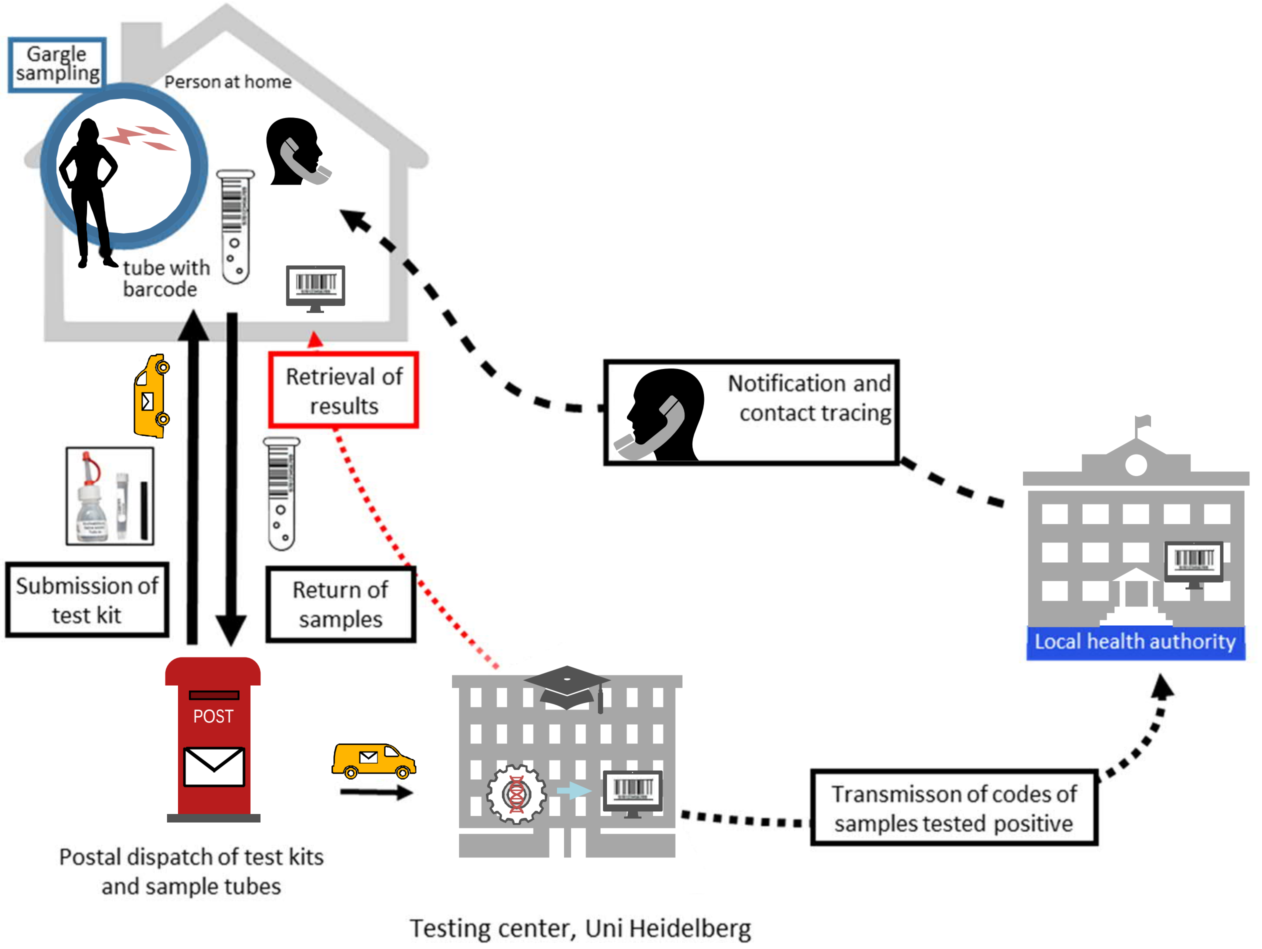

Study materials

VIRUSFINDER
FÜR DICH. FÜR UNS. FÜR ZUKUNFT.

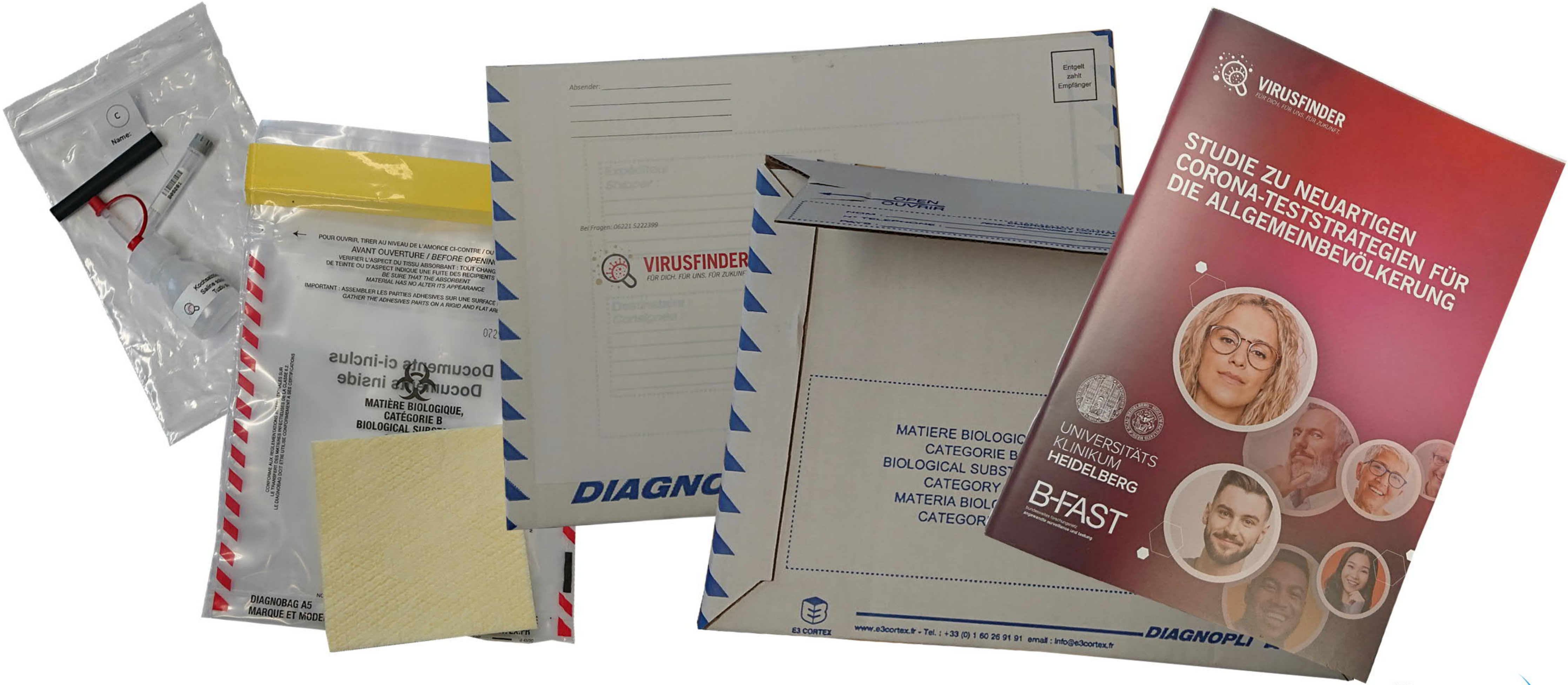

ZMBH

UK HD

Sampling instructions (online available as video)

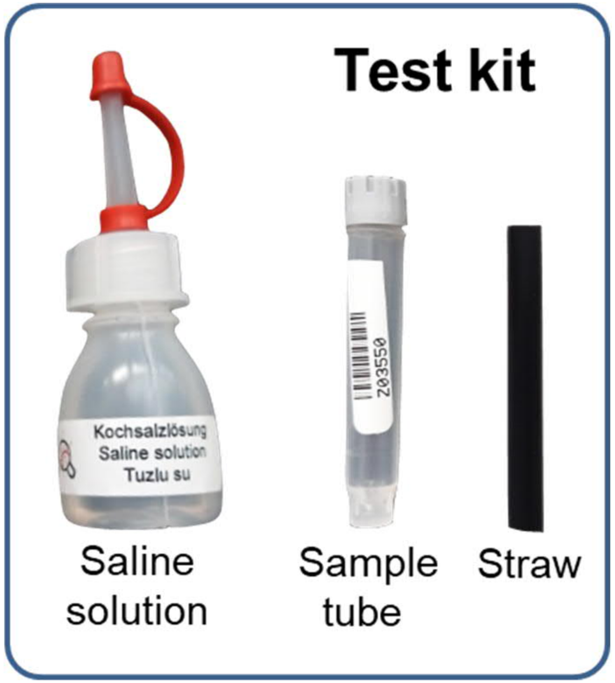

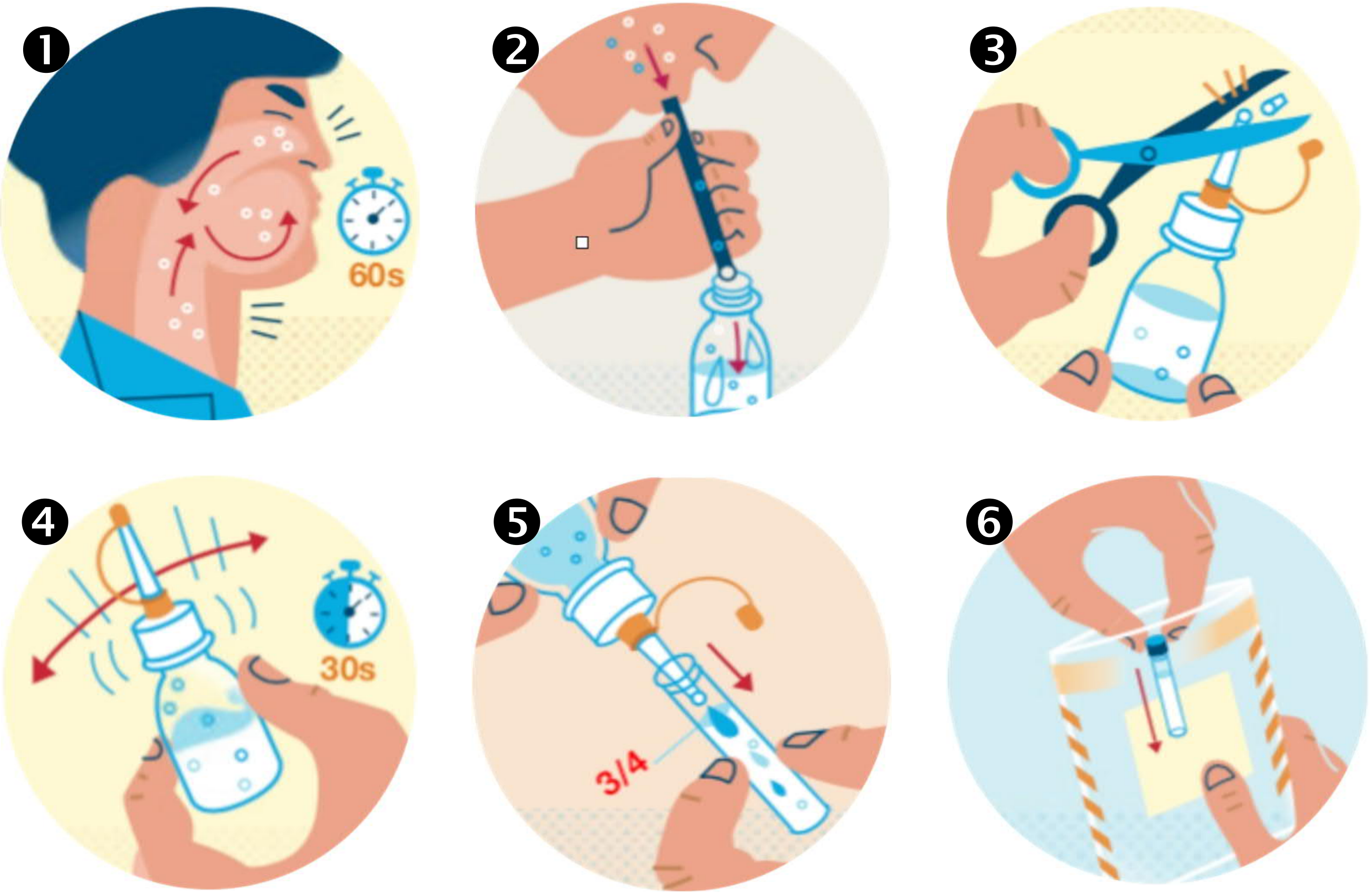

Optimized RT-LAMP sensitivity

VIRUSFINDER
FÜR DICH. FÜR UNS. FÜR ZUKUNFT.

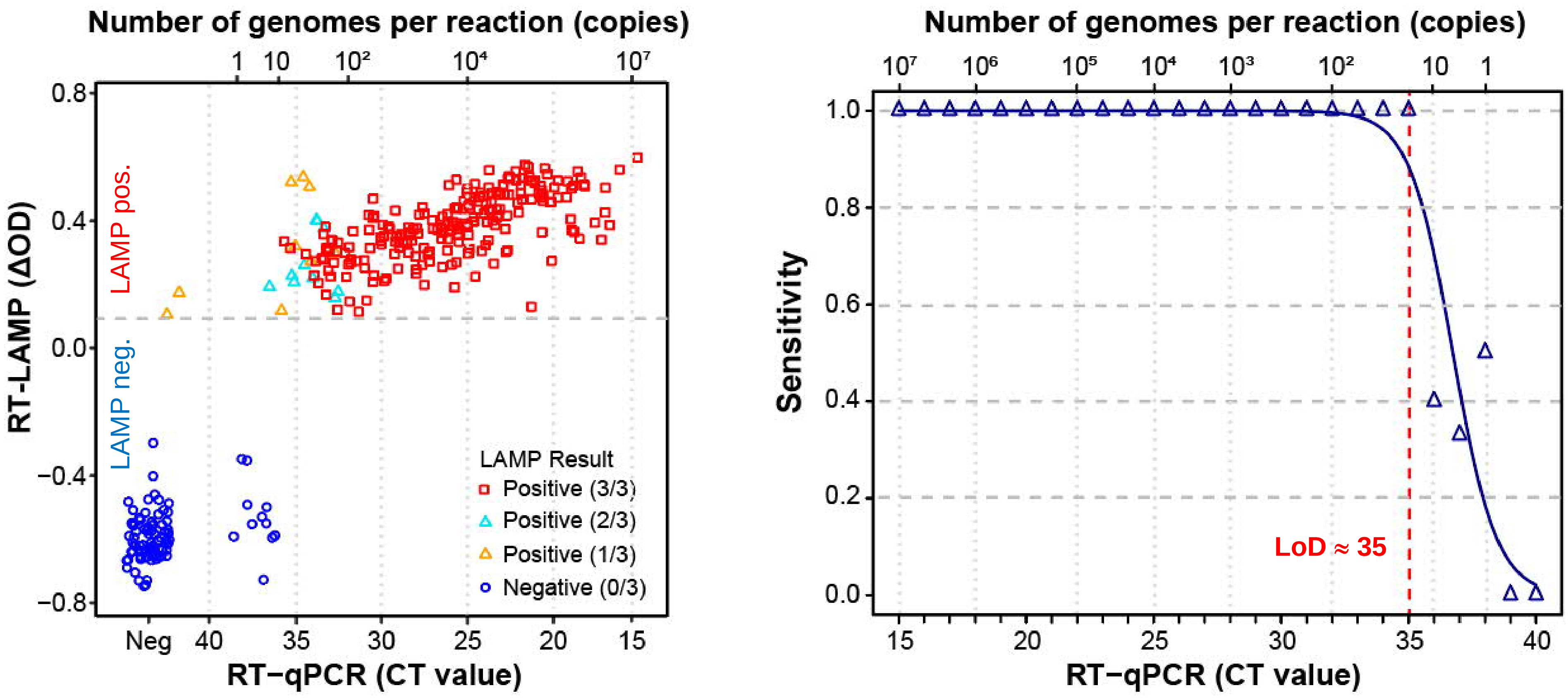

(PCR: TIBMolbiol N/E gene)

Data published in: Lou et al., 2023. Scalable RT-LAMP-based SARS-CoV-2 testing for infection surveillance with applications in pandemic preparedness. *EMBO reports*. 24:e57162. doi:10.15252/embr.202357162. Reproduced in line with the CC-BY 4.0 licence policy.

ZMBH

UKHD

Data sets

- Study sample data (N=27,909; residence, age, sex, study arm, standardized and not normalized study weights)
- Events (N=83,912; logistic events, server events (online requests), laboratory events Labor; each with timestamp)
- Laboratory data (LAMP + PCR)
- Pre-Screening tool (N=10,138; 16 COVID-19 symptoms (current and frequency), disease groups, contact with patients/childrech, household size, occupation, educational attainment)
- Hotline (N=2042 calls, max. 177/day)
- Feedback (N=412): satisfaction with processes/materials (Likert-scale), sampling duration time, free text on suggestions
- Costs (Workload per employee/process step, material costs, personnel costs, etc.)
- Comprehensive interviews (N=85; each around 60 minutes): 15 responders each in study arms A and B, 15 non-responders each in A und B, 15 asymptomatic cases → topped up with University campus tests)

Thank you for listening!

www.virusfinder.de

- Prof. Michael Knop
- Dr. Simon Anders
- Dr. Robin Burk
- Lucia Brugnara
- Konrad Herbst
- Dr. Dan Lou
- Yuanqiang Duan
- Dr. Britta Knorr
- Dr. Aurelia Souares
- Dr. Shannon McMahon
- Prof. Manuela De Allegri
- Dr. Hoa Nguyen
- Dr. Tobias Siems
- Dr. Claudia Denkinger
- Prof. Till Bärnighausen
- Dr. Kathleen Boerner
- Dr. Matthias Sand (GESIS)
- Ralf Fieger (astrals)
- Dr. Andreas Welker
- Matthias Meurer
- Lisa Koeppel
- Dr. Stephan Brenner
- Svetlana Ovchinnikova
- Florian Huber
- Leonhard Hacker
- Victoria Witte
- Max Jacobs
- Prof. Michael Marx
- Dr. Andreas Deckert

u.v.a.

VIRUSFINDER
FÜR DICH. FÜR UNS. FÜR ZUKUNFT.

Some impressions from the logistics…

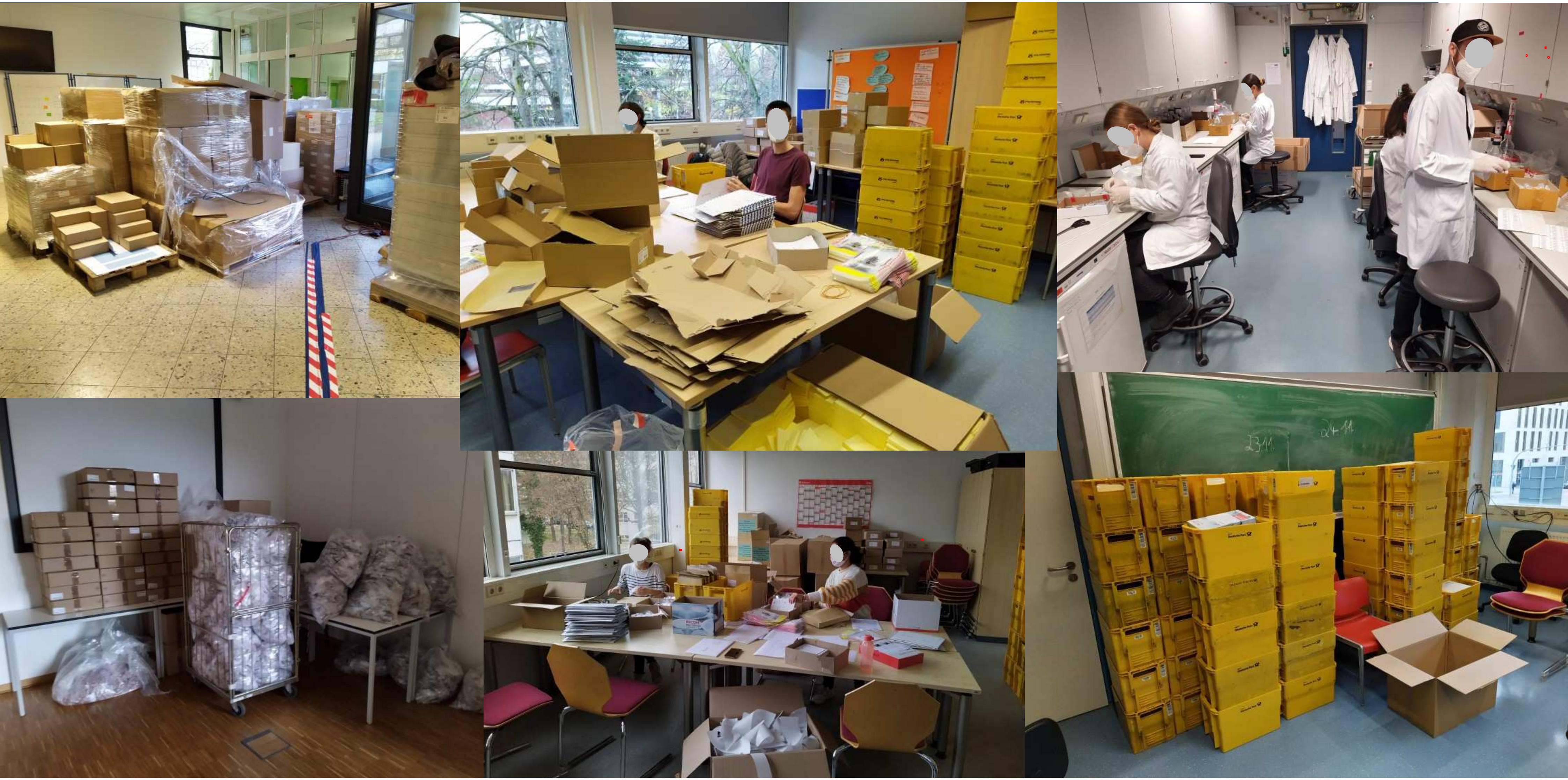

Supplement: Multimedia Appendix 3 [file publichealth_v9i1e44204_app3.pdf]
